# Detection of Genomic Regions Controlling the Antioxidant Enzymes, Phenolic Content, and Antioxidant Activities in Rice Grain through Association Mapping

**DOI:** 10.3390/plants11111463

**Published:** 2022-05-30

**Authors:** Priyadarsini Sanghamitra, Saumya Ranjan Barik, Ramakrushna Bastia, Shakti Prakash Mohanty, Elssa Pandit, Abhisarika Behera, Jyotirmayee Mishra, Gaurav Kumar, Sharat Kumar Pradhan

**Affiliations:** 1 ICAR-National Rice Research Institute, Cuttack 753006, Odisha, India; p.sanghamitra1@gmail.com (P.S.); saumya_bt06@yahoo.co.in (S.R.B.); ramakrushnabastia@gmail.com (R.B.); evenpunk22@yahoo.co.in (S.P.M.); elsambio@gmail.com (E.P.); abhisarika_in@yahoo.com (A.B.); jyotirmayeemishra26@gmail.com (J.M.); gauraviari@yahoo.in (G.K.); 2 Department of Biosciences and Biotechnology, Fakir Mohan University, Balasore 756020, Odisha, India

**Keywords:** antioxidant enzyme, association mapping, catalase, CUPRAC, DPPH, FRAP, peroxidase

## Abstract

Because it is rich in antioxidant compounds, the staple food of rice provides many health benefits. Four antioxidant traits in rice grain, *viz.*, catalase, CUPRAC, DPPH, FRAP and peroxidase, were mapped in a representative panel population containing 117 germplasm lines using 131 SSR markers through association mapping. Donor lines rich in multiple antioxidant properties were identified from the mapping population. The population was classified into three genetic groups and each group showed reasonable correspondence with the antioxidant traits. The presence of linkage disequilibrium in the population was confirmed from the estimated Fst values. A strong positive correlation of DPPH was established with TPC, FRAP and CUPRAC. A moderate to high mean gene diversity was observed in the panel population. Eleven significant marker-trait associations for antioxidant traits were mapped, namely, *qACD2.1*, *qACD11.1* and *qACD12.2* for DPPH; *qCAT8.1* and *qCAT11.1* for catalase; *qFRAP11.1, qFRAP12.1* and *qFRAP12.2* for FRAP; and *qCUPRAC3.1*, *qCUPRAC11.1* and *qCUPRA12.1* regulating CUPRAC. Co-localization of the QTLs for *qACD11.1, qFRAP11.1* and *qCUPRAC11.1* were detected, which may act as antioxidant hotspots regulating DPPH, FRAP and CUPRAC activities, respectively, while *qACD12.2* and *qFRAP12.1* remained close on the chromosome 12. These detected QTLs will be useful in antioxidant improvement programs in rice.

## 1. Introduction

Antioxidants protect the plant cell from damage and act as a defense system for maintaining the structural and functional integrity of cell [1,2]. Antioxidants also influence the seed viability, vigor and longevity by preventing seed deterioration [2,3,4]. Further, several antioxidants of rice have impressive health benefits [5,6,7]. Consumption of whole grain rice enriched with antioxidant compounds improves human health by reducing the risk of a number of chronic diseases [8,9]. Rice is used as a staple food for more than two billion people globally, and enriching grain with antioxidant compounds may be the best option to utilize rice as a health-promoting food. Moreover, improvement in antioxidants will lead to development of superior quality seeds, which will enhance rice production because good seed is a basic and vital input for crop production [10]. In addition, rice seed enriched with antioxidants (catalase and peroxidase) also enhances resilience in rice crop to stress situation [11,12,13,14]. The antioxidant traits are complex, polygenic in nature and quantitatively inherited [15]. There is a need to develop molecular markers for enhancing these phytochemicals in rice through molecular breeding approach.

In rice, ascorbate peroxidase (APx) was reported to be encoded by eight APx genes: two cytosolic isoforms encoded by *OsAPx1* and *OsAPx2*, two peroxisome/glyoxysome isoforms encoded by *OsAPx3* and *OsAPx4*, and chloroplastidic isoforms encoded by *OsAPx5*, *OsAPx6*, *OsAPx7* and *OsAPx8* [13,16]. The catalase (CAT) is encoded by a small family of three genes: CatA, CatB and CatC [13,17]. Reports on suitable marker loci for identifying these genes for catalase (CAT) and peroxidase (PEROX) are not available. However, several reports on QTL mapping are available on total phenolics content (TPC) and activity of antioxidants such as DPPH (2,2’diphenyl picryl hydrazyl), FRAP (ferric reducing antioxidant power) and CUPRAC (cupric reducing antioxidant capacity) [8,10,18,19,20]. For better understanding of these complex traits, more genes/loci need to be identified that will lead to development of trait-specific markers, which will accelerate the efforts to breed high-yielding antioxidant-rich rice varieties. The present study mainly aimed at QTL mapping of antioxidant traits, such as CAT, PEROX, TPC, DPPH, FRAP and CUPRAC activity, in rice.

Association mapping has emerged as a powerful alternative strategy for identifying genes or QTLs for various complex traits in plants in a natural variable population by examining the marker–trait associations. Mapping of complex antioxidant compounds and antioxidant activity by exploiting the naturally occurring variations through association mapping will provide QTLs that regulate the phytochemicals in rice. The genetic diversity and structure of the population in association mapping will be helpful for detecting marker–trait associations that may be useful for trait enhancement in molecular breeding programs. In order to avoid spurious marker–phenotype association, population structure (Q) with relative kinship (K) analyses are used to check and correct the panel population composition for linkage disequilibrium (LD) mapping analyses [21,22,23,24]. The association estimates based on both the generalized linear model (GLM) and mixed linear model (MLM) are considered appropriate for mapping complex traits, and have been shown to perform better than other model analysis. 

In the present investigation, association mapping was performed in a panel population containing 117 genotypes (64 white and 53 red grain) shortlisted through phenotyping of six phytochemical traits (CAT, PEROX, TPC, DPPH, FRAP and CUPRAC) from 270 shortlisted diverse genotypes of India. The population was studied for the population genetic structure, diversity and association of molecular markers with these phytochemical traits.

## 2. Results

### 2.1. Phenotyping of the Population for Antioxidant Traits in Rice

**Figure 1:**
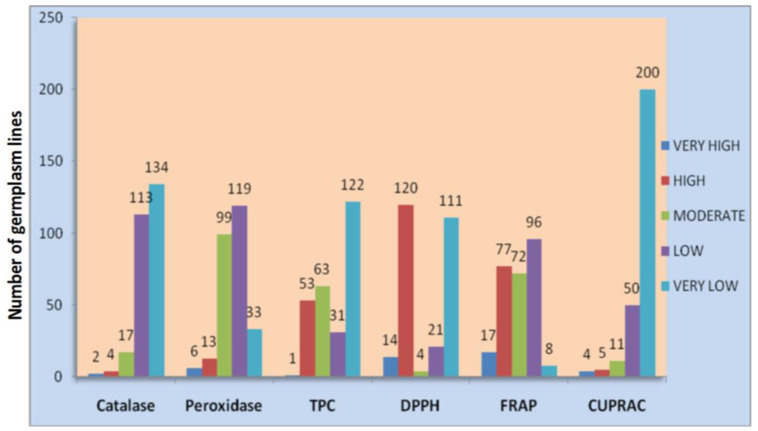
Frequency distribution of genotypes containing each antioxidant trait, namely, catalase, peroxidase, total phenolics content (TPC), 2,2-diphenyl-1-picrylhydrazyl (DPPH), ferric reducing antioxidant power (FRAP) and cupric ion reducing antioxidant capacity (CUPRAC) in the 270 shortlisted germplasm lines.

**Figure 2:**
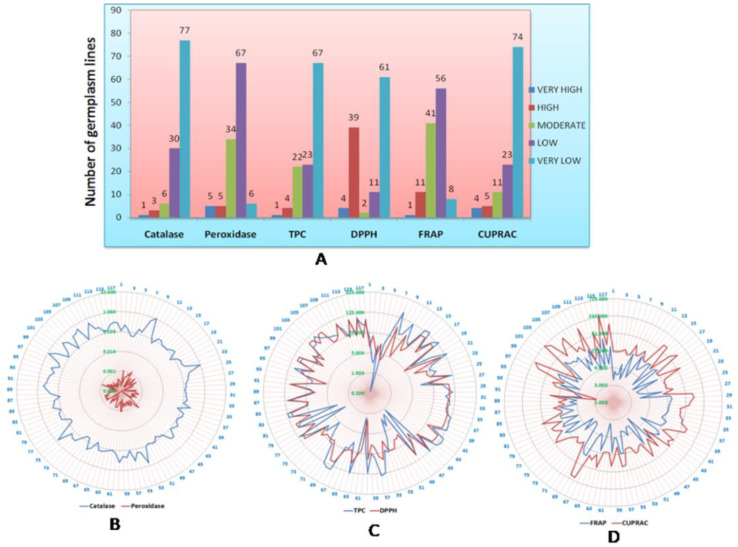
(**A**). Frequency distribution of germplasm lines for antioxidant properties, catalase, peroxidase, total phenolics content (TPC), 2,2-diphenyl-1-picrylhydrazyl (DPPH), ferric reducing antioxidant power (FRAP) and cupric ion reducing antioxidant capacity (CUPRAC) present in the panel population of 117 genotypes. (**B**). Spider graph showing the catalase and peroxidase activities. (**C**). TPC content and DPPH activities. (**D**). FRAP and CUPRAC activities.

A total of five antioxidant traits of rice grain, namely, catalase, peroxidase, DPPH, FRAP and CUPRAC, along with antioxidant compounds and phenolic content, were estimated from 270 genotypes during the wet season of 2018 (Supplementary Table S1). Wide variation was observed for the six antioxidant traits among the germplasm lines. Each antioxidant trait in the population was grouped into five classes and the germplasm lines were classified into various groups (Figure 1). The frequency distribution of each group or population is presented in Figure 1. A representative mapping population for the six antioxidant traits was developed by shortlisting 117 germplasm lines from all groups and traits from the initial 270 germplasm lines (Table 1; Figure 2). The average values of the six antioxidant traits estimated from the panel population exhibited wide variation among the germplasm lines for the studied traits (Table 1). Very high values for catalase content were observed in the germplasm lines AC.9030, Shayam, AC.9093, AC.20282, Sugandha-2, AC.44646, AC.43660 and AC.43732. Moreover, very high peroxidase concentration was estimated from the germplasm lines AC.9028, AC.9035, AC.20845, Latachaunri, AC.10608, AC.7282, AC.9053A and AC.44585. Genotypes such as Kundadhan, AC.9063 and AC.20282 showed higher phenolic content in the seeds of the panel population. The DPPH enzyme activity was found to be the predominant antioxidant enzyme activity present in the population, representing 39.32% of the population. High activity of FRAP was noticed in the seeds of the genotypes, namely, AC.20282, AC.20246, AC.44646, AC.44595, AC.44588 and AC.43660. The potential donors identified from the population containing a high level of CUPRAC for total antioxidant capacity were found in the germplasm lines AC.20282, AC.44646, AC.44595, AC.44588 and AC.43660. However, a higher level of antioxidant properties for more than 2 traits was detected in the germplasm lines Kundadhan, Latachaunri, AC.20282, AC.20246, AC.44646, AC.44595, AC.43737, AC.43660, AC.43732, AC.43738 and AC.43670.

### 2.2. Genotype-by-Trait Biplot Analysis for Relatedness among the Germplasm Lines for the Antioxidant Traits

**Figure 3:**
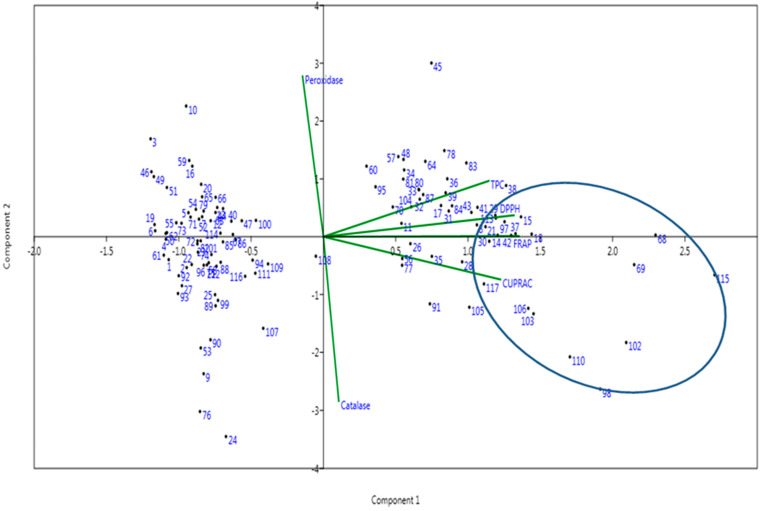
Genotype-by-trait biplot diagram of 117 germplasm lines in the two PCs for the antioxidant traits, catalase, peroxidase, total phenolics content (TPC), 2,2-diphenyl-1-picrylhydrazyl (DPPH), ferric reducing antioxidant power (FRAP) and cupric ion reducing antioxidant capacity (CUPRAC).

The genotype-by-trait biplot diagram was generated using the first two principal components of the six antioxidant properties estimated from the mapping population of the panel containing 117 germplasm lines (Figure 3). A total of 53.14 and 19.57% of the total variability, and eigen values of 3.189 and 1.17, were estimated for the 1st and 2nd principal components, respectively (Supplementary Figure S1). Peroxidase contributed maximum diversity followed by catalase and FRAP among the six antioxidant traits studied using the panel population (Figure 3). The distribution pattern of germplasm lines in the four quadrants of the biplot indicate that genotypes carrying high concentrations of antioxidants are seen in the 1st (top right) and 2nd (bottom right) quadrants. Higher concentrations of multiple antioxidant compounds and enzymes containing germplasm lines are depicted in the circle located in these two quadrants (Figure 3). The top right (1st quadrant) and bottom right (2nd quadrant) quadrants showed the majority of the genotypes containing high antioxidant traits in the germplasm lines. The 3rd (bottom left) quadrant accommodated most of the genotypes with high catalase activity and poor activity in other antioxidants. The 4th quadrant (top left) showed a majority of the germplasm lines that were rich in DPPH activity and poor in other antioxidant enzymes and compounds (Figure 3).

### 2.3. Nature of Association among Antioxidant Traits

**Figure 4:**
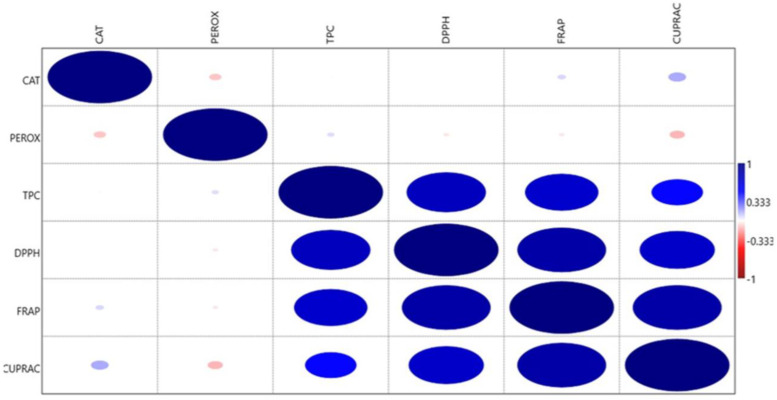
Heat map showing Pearson’s correlation coefficients for the antioxidant traits. Significant correlations are colored either red (negative) or blue (positive). Shades of blue indicate increasing positive correlation coefficient; shades of red indicate increasing negative correlation coefficient. CAT: catalase (unit min ^−1^ g^−1^); PEROX: guaiacol peroxidase (unit min ^−1^ g^−1^); TPC: total phenolics content (mg catechol or CE 100 g^−1^); DPPH: 2, 2-diphenyl-1-picrylhydrazyl (% inhibition); FRAP: ferric reducing antioxidant power activity (µg ascorbic acid equivalent or AAE) g^−1^; CUPRAC: cupric ion reducing antioxidant capacity (μg trolox equivalent or TE) g^−1^).

The association among six antioxidant traits revealed a strong positive correlation (r ≥ 0.7) of DPPH with TPC, FRAP and CUPRAC. Moreover, a strong correlation was noticed in FRAP with CUPRAC (Figure 4). However, TPC showed a moderate positive correlation (r: 0.5–0.7) with FRAP, but with CUPRAC a weak positive correlation (r < 0.5) was observed. Although CAT and CUPRAC showed a negative correlation with PEROX, the association was not significant (Figure 4). These positively or negatively correlated antioxidant traits may be controlled by the closely linked genes or because they may be structurally related. Therefore, a variety that accumulates high concentrations of one antioxidant may contain higher quantities of other correlated antioxidants.

### 2.4. Genetic Diversity Parameters Analysis

The representative mapping population containing 117 germplasm lines that exhibited wide variation for the six antioxidant traits was genotyped using 131 SSR markers. The genetic diversity parameters assessed from the panel population are shown in the Supplementary Table S2. The population showed a total of 508 marker alleles with mean alleles of 3.74 per locus detected by the genotyping results using 131 SSR markers. The number of detected alleles varied from 2 to 7 per marker per locus. The highest number of alleles was produced by the marker RM493 in the studied population for antioxidant contents in rice. The major allele frequency parameter was used for detection of variation by a marker in the population. The average major allele frequency linked to the polymorphic markers was computed to be 0.5598, with a range of 0.282 (RM493 and RM8044) to 0.923 (RM6054) (Supplementary Table S2). Informative genetic markers were detected by the PIC values. These showed a range of 0.142 (RM6054) to 0.789 (RM493), with a mean value of 0.496. The mean heterozygosity (Ho) observed from the population was 0.111, and heterozygosity varied from 0.00 to 0.957 (RM3735). A total of 23 marker loci showed a heterozygosity (Ho) value of 0.00 in the panel population. The mean gene diversity (He), which gives a measure of genetic diversity in the panel population, was 0.556, and gene diversity varied from 0.145 (RM6054) to 0.814 (RM493).

### 2.5. Population Genetic Structure Analysis

STRUCTURE 2.3.6 software was used for analysis of genetic structure by applying probable subpopulations (K) at a higher delta K-value in the used diverse population for the six antioxidant traits. The rate of change in the log probability of data between successive K values was the delta K value used in the analysis. The panel population was classified into two subpopulations by assuming K = 2 and using a ∆K peak value of 309.09 (Supplementary Table S3; Supplementary Figure S2). The two subpopulations were in the proportions of 0.727 and 0.273 for population 1 and population 2, respectively. The two subpopulations showed correspondence with antioxidant traits carrying genotypes present in the studied population; however, many germplasm lines were also included that did not show a correspondence with the six antioxidant traits. Therefore, the next ∆K peak value of 81.79 at K = 3 was compared, in which the population was classified into three subpopulations, and each subpopulation showed a reasonable correspondence with the majority of the members, and comparatively better correspondence than at K = 2 for the correlation of the germplasm lines with the six antioxidant traits. The three subpopulations showed proportions of the inferred cluster value of 0.69, 0.206 and 0.104 for the subpopulations 1, 2 and 3, respectively. The Fst1, Fst2 and Fst3 values were 0.168, 0.356 and 0.362 for the subpopulations 1, 2 and 3, respectively (Supplementary Table S3; Supplementary Figure S3). The ancestry value of ≥80% obtained in a genotype categorized the genotype into the particular subpopulation.

**Figure 5:**
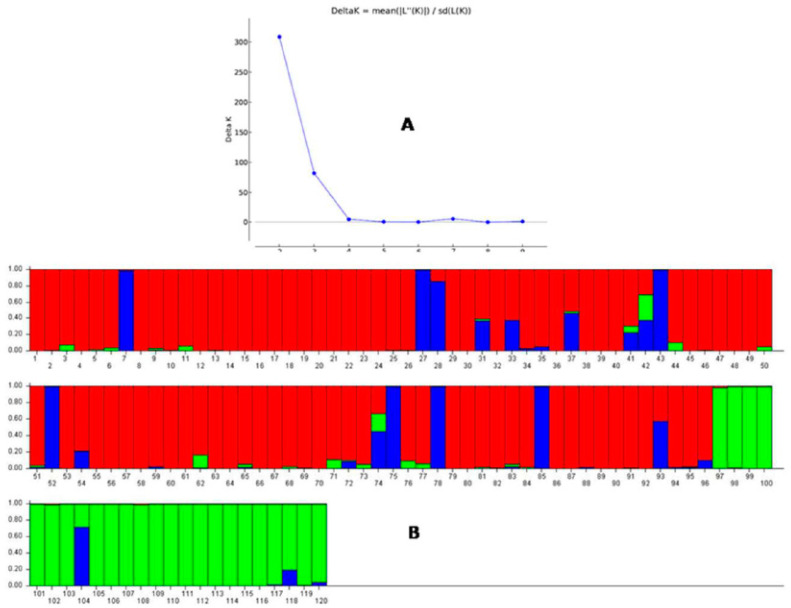
(**A**) Graph of ∆K value against the rate of change in the log probability of data between successive K values. (**B**) Population structure of the panel population based on membership probability fractions of individual genotypes at K = 3. The genotypes with the probability of ≥80% of membership proportions were assigned as subgroups whereas others were grouped as the admixture group. The numbers in the diagram depict the serial number of the germplasm lines listed in Table 1.

The majority of the germplasm lines with high to very high antioxidant traits were present in the subpopulation 2. The moderate value antioxidant properties containing germplasm lines were present in the subpopulation 3. The majority of the germplasm lines showing poor to moderate values for antioxidants were in subpopulation 1 (Table 2; Figure 5). A low alpha value (α = 0.0441) was estimated for the panel population by the structure analysis at K = 3. Positively skewed leptokurtic distributions were observed for the mean alpha value, whereas normally skewed leptokurtic distributions detected for each of the three Fst values of the three subpopulations showed a distinct variation in the distribution among the Fst values (Supplementary Figure S4).

### 2.6. Molecular Variance (AMOVA) and LD Decay Plot Analysis

Plants related by ancestry or by traits in a population are grouped into different population structures. The genetic variations within and between the subpopulations were computed at K = 3 for the analysis of molecular variance (AMOVA) (Table 3). The genetic variation among the populations estimated at K = 3 was computed to be 1%; among individuals it was 4%, and there was 95% variation within individuals in the panel population. Wright’s F statistic was used to determine the deviation from Hardy–Weinberg’s prediction. The parameter F_IT_ for individuals within the total population for differentiation and F_IS_ for the uniformity of individuals within the subpopulation in a population were computed. The F_IS_ and F_IT_ values within the population and the total population estimated on the basis of 131 marker loci were 0.045 and 0.051, respectively, whereas the total population had an F_ST_ value of 0.006 between the three subpopulations. Fst is used to indicate the subpopulations or population differentiation within the total population. A clear differentiation between the three subpopulations was observed from their distribution pattern based on the Fst values (Supplemental Figure S4).

**Figure 6:**
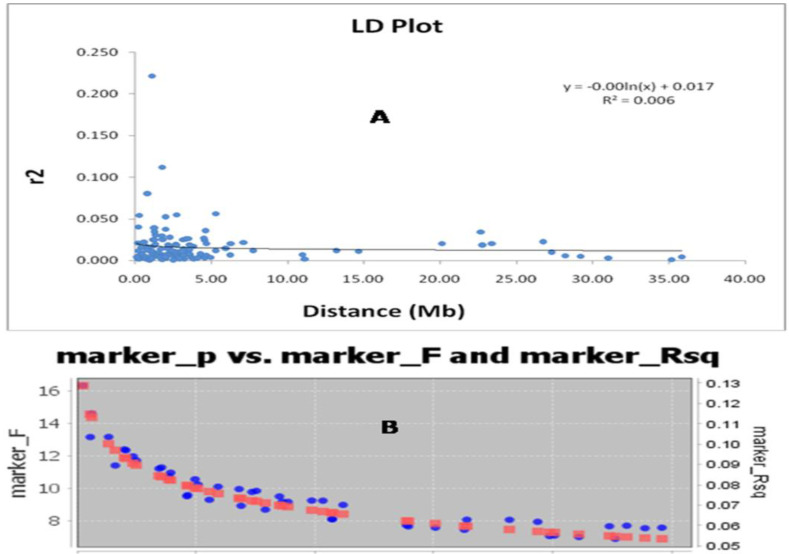
(**A**) The physical distance (megabase pairs, Mb) between pairs of loci on chromosomes against the linkage disequilibrium (LD) decay (*R*^2^) curve plotted in rice. The decay in million bp was estimated by taking the 95th percentile of the distribution of *R*^2^ for all unlinked loci. (**B**) The marker ‘P’ versus marker ‘F’ and marker *R*^2^.

The association of alleles by different loci in a nonrandom manner is utilized in the marker–trait association analysis. The existence of marker–trait association is dependent on the LD decay rate in a population over a time period. The LD decay rate indicates the possibility of new genes or allelic variants controlling the antioxidant compounds associated with molecular markers for these traits. The syntenic *r*^2^ value was used to plot the linkage disequilibrium decay of the population versus the physical distance in million base pairs (Figure 6A). Tightly linked markers had a higher *r*^2^ value and the average *r*^2^ values rapidly decreased as the linkage distance increased. In the LD plot, it is observed that the LD decay in the beginning was delayed in the studied panel populations. However, a decline in the LD decay can be noted in the curve for the associated markers at about 1–2 megabase pairs and, thereafter, a gradual and very slow decay can be noted. The graph clearly indicates the continuance of linkage disequilibrium decay in the population for the studied antioxidant properties in the rice population. The limitation for the LD decay depends on non-random mating, mutation, selection, migration or admixture, and genetic drift influences the estimates of LD. This LD decay plot also provides clues about the creation of genetic admixture groups for various antioxidant compounds in the normal population. A similar trend was also noted in the marker ‘P’ versus marker ‘F’, and the marker R^2^ (Figure 6B) curve. The detected markers from this study indicate the strength of the markers for the studied antioxidant traits. 

### 2.7. Principal Coordinates and Cluster Analyses for Genetic Relatedness among the Germplasm Lines

**Figure 7:**
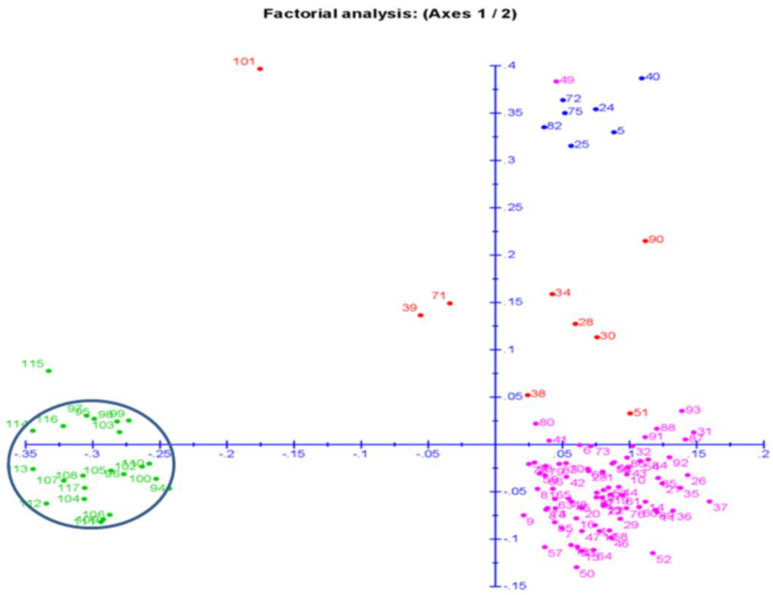
Distribution of 117 genotypes for the principal coordinate analysis (PCoA) based on 131 molecular markers’ genotyping for the six antioxidant traits. The dot numbers in the figure represent the serial number of the genotypes listed in Table 1. The numbers are colored on the basis of the subpopulations obtained from the structure analysis (SP1: pink; SP2: green; SP3: blue; Admix: red).

The two-dimensional plot for principal coordinate analysis (PCoA) was constructed based on the genotyping results of 131 SSR markers, and was used to classify the 117 germplasm lines as per the genetic relatedness among the lines (Figure 7). The inertia shown by component 1 was 12.29%, whereas component 2 exhibited 7.35%. The germplasm lines were assigned to the four quadrants at different places, forming three major groups (Figure 7). The biggest group accommodated all the germplasm lines of the subpopulations 2 and 3, and was clustered in the 2nd (bottom right) quadrant. The genotypes in the 1st quadrant were divided into two groups, of which one group on the top of the 1st quadrant form the SP3 subpopulation, which has low to very low antioxidant properties in the seeds. The other group near to axis 1 comprises only the admix type of germplasm lines. Several germplasm lines of quadrant 2 and those closer to axis 1 are also admix genotypes. The admix genotypes present on both sides of axis 1 are depicted in red (Figure 7).

The germplasm lines containing high to very high mean values for the antioxidant traits are grouped together, forming the subpopulation 3. This subpopulation is present on quadrants 3 (top left) and 4 (bottom left), and are encircled in blue. The germplasm lines rich in the antioxidant properties are placed on both sides of the axis on quadrants 3 and 4 (Figure 7). The PCoA distributed all the germplasm lines into the four quadrants, classifying them into four clusters and a separate admixture group. The subpopulations clustered by PCoA showed correspondence with the population structure (Figure 7). Germplasm lines Ac. 44594, Ac. 43669, Ac. 44597, Ac. 44588, Ac. 43737, Ac. 44595, Ac. 43676, Ac. 44597, Ac. 44592, Ac. 43738 and Ac. 44646 are placed together in one structural group present in quadrants III and IV, and are rich in the antioxidant traits. The PCoA placed germplasm lines in quadrant II for which antioxidant properties were mostly at average levels. This quadrant formed the group comprising all of the germplasm lines of subpopulations 1 and 2.

**Figure 8:**
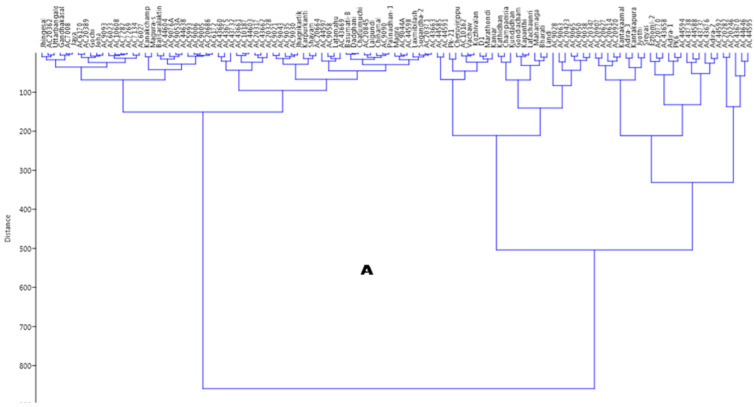

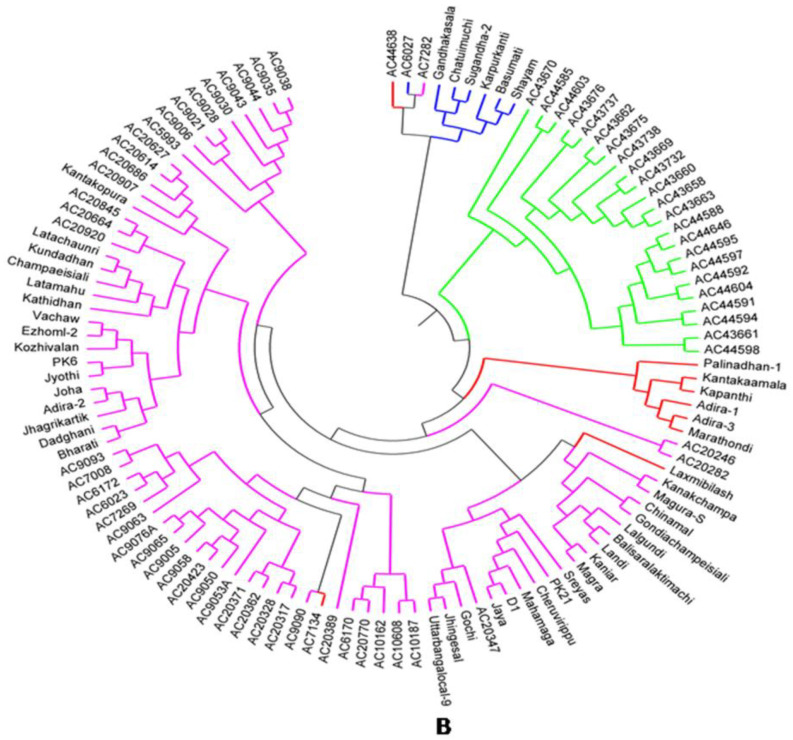
(**A**) Grouping of the germplasm lines present in the panel population by Ward’s clustering based on the six antioxidant traits. (**B**). Unrooted tree using unweighted-neighbor joining method depicting clustering patterns of 117 germplasm lines genotyped by 131 molecular markers colored on the basis of the subpopulations obtained from structure analysis (SP1: pink; SP2: green; SP3: blue; Admix: red).

Ward’s clustering approach broadly grouped all the genotypes into two major groups. The largest cluster, cluster II accommodated 65 germplasm lines that all carried very low, low or medium levels of antioxidant properties. Cluster I only had 52 germplasm lines. The dendrogram placed in this cluster all the germplasm lines that were rich in antioxidant traits for at least one compound. This cluster was again subdivided into two subgroups, which were further divided into sub-subclusters. Cluster II was divided into two main subclusters, which were finally divided into small groups. All of the sub-subclusters accommodated in Ward’s clustering approach were based on the antioxidant traits present in the germplasm lines (Figure 8A).

The cluster analysis differentiated the germplasm lines on the basis of genotyping of 131 SSR markers and placed the genotypes into different clusters that corresponded with the studied antioxidant traits. The unweighted-neighbor joining tree differentiated the genotypes into three different clusters (Figure 8B). The cluster for subpopulation 3 was differentiated from SP_2_ by the presence of germplasm lines containing high antioxidant properties, whereas moderate to high containing genotypes were placed in subpopulation 2. The green-colored portion of the tree is designated SP2 whereas SP3 is shown in blue. The very poor antioxidant properties carrying germplasm lines were in subpopulation 3. The majority of the germplasm lines present in subpopulation 1 were poor to medium in antioxidant traits and are shown in pink. The germplasm lines with an admix type of population are depicted in red in the neighbor joining tree (Figure 8B).

### 2.8. Marker–Trait Association for Antioxidant Traits in Rice

**Figure 9:**
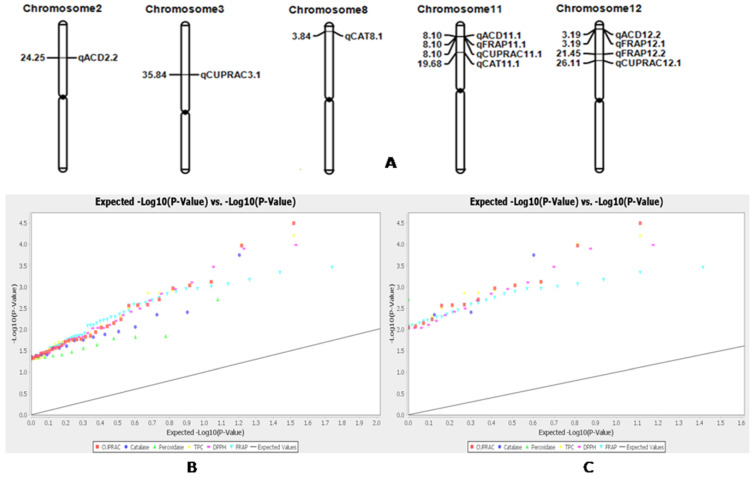
(**A**) Positions (Mb) of the QTLs on the chromosomes regulating the antioxidant traits, catalase, peroxidase, DPPH, FRAP and CUPRAC detected by association mapping in rice. B. Distribution of marker–trait association and quantile–quantile (Q–Q) plot generated by generalized linear model analysis for the six antioxidant traits at (**B**) *p* < 0.05 and (**C**) at *p* < 0.01.

Marker–trait associations for total phenolic content; catalase and peroxidase for antioxidant enzymes; DPPH and FRAP for antioxidant activities; and CUPRAC for antioxidant capacity were computed by using the generalized linear model (GLM) and mixed linear model (MLM/ K+Q model)) in the TASSEL 5 software. The marker–trait association values were compared at less than 1% error, i.e., 99% confidence (*p* < 0.01). Five traits showed significant association with 43 SSR markers by GLM, and four traits with 14 SSR markers by MLM analysis at *p* < 0.01. The marker R^2^ values varied from 0.05438 to 0.12875 by GLM, and from 0.06324 to 0.12586 by the mixed linear model (Supplementary Tables S4 and S5). A total of 11 significant marker–trait associations were detected by both the models for four antioxidant traits at *p* < 0.01 in the seeds of the germplasm lines (Figure 9A). Three significant marker–trait associations were detected for each of the traits, DPPH, FRAP, CUPRAC, and catalase (Table 4; Figure 9A). The Q–Q plot also confirmed the association of these markers with the associated antioxidant traits in rice (Figure 9B).

Two markers, namely, RM1341 and RM3231 showed significant associations with the antioxidant enzyme, catalase, analyzed by GLM and MLM models at *p* < 0.01, and were present on chromosomes 11 and 8, respectively. The QTLs controlling the antioxidant activity of FRAP showed an association with SSR markers RM247 and RM309 present on chromosome 12. RM3701, which was present on chromosome 11, also showed a significant association with FRAP in both of the models. The CUPRAC assay was found to be significantly associated with marker RM235 present on chromosome 12 at the 101.8 cM position. Moreover, the enzyme was strongly associated with RM148 located at 142.3 cM on chromosome 3, as detected by both the models. The antioxidant activity measured by DPPH was found to be significantly associated with the markers RM247 and RM3701 present on chromosomes 12 and 11, respectively (Table S5; Figure 9A). The Q–Q plot also confirmed the associations of these markers with the estimated antioxidant traits in rice (Figure 9B).

The association mapping study for the antioxidant traits in rice seeds identified co-localization of QTLs controlling antioxidant properties in rice. It is observed that the same markers showed significant associations with different antioxidant traits in rice in both of the models (Table 4). Significant associations of marker RM3701 with antioxidant activities of DPPH, CUPRAC and FRAP present in the germplasm lines were detected. In addition, the association of RM247 with antioxidant activity of DPPH and FRAP was also detected by both of the models at <1% error and *p* < 0.01 (Table 4). In analysis of marker association by GLM, the markers RM468 and RM167 showed associations with both DPPH and FRAP activities.

## 3. Discussion

Rice is the staple food for the majority of the world population. Many antioxidant compounds and enzymes are present in different rice germplasm lines that provide health benefits. Mapping of these genes for regulating the antioxidant traits in rice germplasm lines and their deployment in breeding programs are very important for enhancing the content in rice grains. The germplasm lines shortlisted for this study showed variation among the lines for the antioxidant traits in the population (Supplementary Table S1; Table 1). The results showed that few antioxidant traits showed correlation among them and will be useful for simultaneous transfer of multiple antioxidant traits into the popular varieties. Therefore, there are possibilities for improvement of the antioxidant enzymes (such as catalase and peroxidase), antioxidant activities (namely, DPPH and FRAP) and antioxidant capacity (by CUPRAC assay), in rice based on the results from genetic variation and correlation obtained from the population (Table 1; Table 3). A number of studies on the existence of genetic variations for antioxidant content and activities have also been reported by researchers [25,26,27,28]. In addition, clear groups and subgroups were obtained in the phenotyping and molecular diversity analyses of antioxidant enzymes present in the population (Supplementary Table S2). The SSR markers showed better PIC values and related diversity parameters in the studied population; this result will be useful in antioxidant improvement programs. The germplasm lines used in this mapping study were from the rice reported for high diversity areas, including the Jeypore region, the secondary center of the origin of rice [29].

Several germplasm lines were identified as potential donors in which more than two traits for antioxidant enzymes and high activities were observed in the seeds. The genotypes rich in multiple antioxidant traits were Kundadhan, Latachaunri, AC.20282, AC.20246, AC.44646, AC.44595, AC.43737, AC.43660, AC.43732, AC.43738 and AC.43670, which will be useful as donors in antioxidant improvement programs (Table 1; Supplementary Table S3). Therefore, inclusion of the panel population for mapping of antioxidant traits will be effective. Structure analysis grouped the population into three subpopulations with different Fst values for each genetic group. The existence of genetic groups supported the continuance of linkage disequilibrium groups in the population. Detection of a moderate alpha value and the existence of many genetic admix-type germplasm lines in the population indicated that these antioxidant traits initially evolved from a single source during evolution of the trait. Different antioxidant compounds, enzymes and activities were formed in admix genotypes with different ancestry values during evolutionary process. A good correspondence of genetic structure group and different traits was found earlier by many researchers [10,30,31,32,33,34,35,36].

Four antioxidant traits were found to be significantly associated with 11 SSR markers analyzed by both GLM and MLM approaches (Table 4). The marker–trait associations detected by both the models at *p* < 0.01 and low p-value are considered to indicate very strong associations, and the markers will be useful in improvement programs. The strongly associated SSR markers—namely, RM1341 and RM3231 for catalase enzyme; RM247, RM309 and RM3701 for FRAP activity; RM235 and RM148 for CUPRAC assay; and RM247 and RM3701for DPPH activity—may be useful markers in marker-assisted antioxidant enzyme improvement programs in rice (Table 4). The Q–Q plot also confirmed the associations of these markers with the antioxidant compounds in rice (Figure 9B).

The QTLs for antioxidant capacity, i.e., DPPH, were reported by earlier researchers in rice [18,19,20]. In this investigation, the marker–trait associations for DPPH were detected with RM247, RM3701 and RM13600 present on chromosomes 12, 11 and 2, respectively. As previously report for the QTL, *qACD12* on chromosome 12 was at the 252.06 Mb location [19]. We detected the QTL associated with marker RM247 at 31.85 Mb, which is a different locus present on chromosome 12. The detected QTL on this chromosome is a new locus and designated as *qACD12.2*, which regulates the DPPH activity in rice seeds. The QTL *qACD2*, reported on chromosome 2 by Shao et al. [19], was at 54.16 Mb. In our investigation, we detected the QTL on chromosome 2 near the location of 242.46 Mb for the trait. The associated QTL on this chromosome may be a new locus and designated as *qACD2.2*, which influences the DPPH activity in rice seeds. Another mapping study for DPPH reports the QTL on chromosome 7 [18]. In addition, the mapping publication of Xu et al. [20] for the trait reports the QTL on chromosome 11. However, in this investigation, a QTL was detected on chromosome 11, which is in contrast to the above-reported QTLs. Therefore, this may be a new locus that regulates antioxidant activity, DPPH, and is designated as *qACD11.1*.

The markers RM1341 and RM3231 were significantly associated with the antioxidant enzyme, catalase, as detected by both GLM and MLM analyses. The locations of these two markers are chromosomes 11 and 8 at 80.2 and 32.7 cM, respectively. No genes or QTLs were reported previously near to this location for the enzyme catalase, and hence the two QTLs are designated as *qCAT11.1* and *qCAT8.1*, respectively. Three markers, namely, RM247, RM3701 and RM309, showed significant association with antioxidant activity, FRAP. The markers positions of RM247 and RM309 are chromosome 12 at 31.85 and 214.54 Mb, respectively. The other marker, RM3701, is located on chromosome 11 at 81.001 Mb. QTL regulating the FRAP activity was not reported near to these locations on chromosomes 11 and 12, or any other regions or chromosomes, in earlier publications. These QTLs controlling FRAP are designated as *qFRAP12.1* near marker RM247 and *qFRAP12.2* near RM309 on chromosome 12, and *qFRAP11.1* on chromosome 11. The antioxidant enzyme, CUPRAC, is detected to be associated with three markers RM3701, RM235 and RM148 on chromosomes 11, 12 and 3 at 81.001, 261.07 and 358.35 Mb, respectively. No QTLs for antioxidant capacity, CUPRAC, were reported earlier in these positions on the chromosomes 3, 11 and 12, or any other regions or chromosomes. Therefore, these three detected QTLs that influence CUPRAC are new loci and designated as *qCUPRAC3.1*, *qCUPRAC11.1* and *qCUPRAC12.1*, respectively.

In this investigation, more than two significant associations were observed in one location analyzed by both the models at <1% error and *p* < 0.01. QTLs present on these locations will be useful for the simultaneous transfer of a greater number of traits. QTLs regulating the antioxidant activities for the assay of DPPH, FRAP and CUPRAC were detected to be co-localized on chromosome 11 near the region 81.001 Mb. This region on chromosome 11 may be considered as an antioxidant hotspot for regulating the activity in rice. Moreover, QTLs for DPPH and FRAP are co-localized on chromosome 12 at the 31.85 Mb position, which is also a hotspot on this chromosome for antioxidant activities (Table S5). These observations of the co-localized candidate genes on the chromosome indicate the usefulness for simultaneous inheritance of these QTLs during improvement programs. Hence, improvement for QTLs regulating the antioxidant enzymes will be very effective in such breeding programs. Recent publications also suggested easy improvements in the co-localized genes controlling various traits in rice [23,24,36,37]. Results of the present investigation showed that association mapping is an effective method to detect more potential loci for antioxidant enzymes and compounds in rice. Additional fine mapping of the detected loci will be undertaken for application in maker-assisted breeding for improvements in antioxidants in rice.

## 4. Materials and Methods

### 4.1. Seed Material

The study materials, consisting of 270 germplasm lines, comprising white (121) and colored (149) rice grain landraces and cultivars, were obtained from Gene Bank, ICAR-NRRI, Cuttack, and grown in the experimental plot of the Institute during the wet season, 2018 (Supplementary Table S1). The initial population was shortlisted on the basis of maturity duration (up to 135 days) and kernel color (red, black, purple and white) from about 1000 germplasm lines. The genotypes were grown in a randomized complete block design in three rows each with spacing of 20 × 15 cm in three replications following a recommended package of practices. Each replication was divided into 5 blocks accommodating 54 germplasm lines in each block. Panicles from the middle row of each genotype and replication were harvested, sun dried for 4–5 days to reduce the moisture content to 11–12%, stored for three months to remove dormancy and then used for estimation of CAT, PEROX, TPC, DPPH, FRAP and CUPRAC activity. A representative panel population containing 117 germplasm lines (white grain: 64; red grain: 53) was shortlisted from the initial 270 shortlisted germplasm lines. The panel population was raised during wet seasons of 2019 and 2020, and the antioxidant traits were estimated. The panel population (117) was used for mapping of antioxidant traits (Table 1).

### 4.2. Phenotyping for the Antioxidant Traits

The seed samples were dehulled by a Satake rice huller, Japan, ground into flour by a grinding machine (Glenmini grinder), sieved through 100-size mesh and stored at 4 °C for the experiment. Seed enzymatic antioxidants such as catalase (CAT: unit min^−1^ g^−1^) and guaicol peroxidase (PEROX: unit min^−1^ g^−1^) were estimated as per the procedures of Aebi [38] and Putter [39], respectively. Non-enzymatic antioxidants such as total phenolics content (TPC) were determined by the modified protocol of Zilic et al. [40] and expressed as catechol equivalent (mg CE100 g^−1^). DPPH (2,2-diphenyl-1-picrylhydrazyl) radical scavenging assay was estimated according to the method of Zhou et al. [41] with little modification, and expressed as % inhibition. Ferric reducing antioxidant power (FRAP) activity was measured as per the modified procedure of Mau et al. [42] and results were expressed as µg ascorbic acid equivalent (AAE) g^−1^. Cupric ion reducing antioxidant capacity (CUPRAC) was determined according to the method of Apak et al. [43] and the result was expressed as μg trolox equivalent (TE) g^−1^.

#### 4.2.1. Statistical Analysis

Cropstat software 7.0. was used for analysis of variance (ANOVA) for each trait, including the estimation of mean, range and coefficient of variation (CV %). Pearson’s correlation coefficients were analyzed to determine the relationship among the various antioxidant traits based on the mean values of the 117 genotypes, and presented in a correlation matrix heatmap using PAST3 software. The germplasm lines were classified into five groups, i.e., very high, high, medium, low and very low categories, based on the mean values of the antioxidant traits.

#### 4.2.2. Genomic DNA Isolation, PCR Analysis and Selection of SSR Markers

Fifteen-day-old plants were used for extraction of genomic DNA from the germplasm lines by adopting the CTAB method [44]. A total of 131 simple sequence repeat (SSR) rice markers across the 12 chromosomes were taken from the database (http://gramene.org/) available in the public domain (Supplementary Table S2). The DNA fragments were resolved in gel electrophoresis for quantification of isolated DNA. PCR analysis was performed using the markers selected based on positions covering all the chromosomes to illustrate the diversity and to identify the polymorphic loci among the 117 rice germplasm lines (Table 1). The conditions of reaction were set to an initial denaturation step (4 min, 94 °C), followed by 35 cycles of denaturation (30 s, 95 °C) and annealing (45 min, 55 °C), extension (1.3 min, 72 °C), final extension (10 min, 72 °C) and storage at 4 °C (infinity). The PCR products were electrophoresed using 3% agarose gel containing 0.80 g ml^−1^ ethidium bromide. A 50 bp DNA ladder was used to determine the size of amplicons. The gel was run up to 4 h at 2.5 V cm^−1^ and photographed using a Gel Documentation System (SynGene). Earlier publications of Barik et al. [45,46] and Pradhan et al. [47] were followed for DNA isolation, electrophoresis and imaging techniques.

### 4.3. Molecular Data Analysis

The presence or absence of amplified products obtained on the basis genotype-primer combination was used to score the data. A binary data matrix was used as discrete variables for the entry of the resulting data. The parameters, namely, polymorphic information content (PIC), observed heterozygosity (H), number of alleles (N), major allele frequency (A) and gene diversity (GD), for each SSR locus were analyzed using ‘Power Marker Ver 3.25’ software [48]. The Bayesian model-based clustering software, STRUCTURE 2.3.6, was used to analyze the genetic data and for population structure [49]. STRUCTURE software was run with K varying from 1 to 10, with 10 iterations for each K value to derive the ideal number of groups. A high throughput parameter set of a burn-in period of 150,000, followed by 150,000 Markov Chain Monte Carlo (MCMC) replications, were adapted during the running period. The highest value of ΔK was obtained from Evanno table used to detect the subpopulation groups from the panel of populations in the next step. The maximal value of L (K) was identified using the exact number of subpopulations. The model choice criterion to detect the most probable value of K was ΔK, and an ad hoc quantity related to the second-order change in the log probability of data with respect to the number of clusters inferred by STRUCTURE was adopted [50]. For estimation of the ΔK value which is function of K, a clear peak was determined as the optimal K value [51]. Structure Harvester was used. The principal coordinate analysis of all the genotypes and unweighted neighbor joining unrooted tree for NEI coefficient dissimilarity index [52] with bootstrap value of 1000 were undertaken using DARwin5 software [53]. Analysis of molecular variance (AMOVA) using GenAlEx 6.5 software was used to estimate the presence of molecular variance across the whole population, within a population and between the subpopulation structures (F_IT_, F_IS_, F_ST_), calculated by the deviation from the Hardy–Weinberg expectation. The procedures followed in earlier publications were adopted for molecular data analysis [23,30,54,55].

To analyze the marker–trait association for mapping study of the seed antioxidant traits in rice, the software “TASSEL 5.0” was used. The generalized linear model and mixed linear model in TASSEL 5.0 were used to determine the genetic association between the phenotypic traits and molecular markers obtained in the study [56]. By considering the significant *p*-value and r^2^ value detected by both the models, the associated markers were identified. The associations of markers were further confirmed by the Q–Q plot generated by the software. The linkage disequilibrium plot was obtained using the LD-measured r^2^ between pairs of markers, and plotted against the distance between the pairs. In addition, the accuracy of the marker–trait association was checked by estimating the FDR-adjusted *p*-values (*q*-values) using R software, as described in the earlier publications [23,30]. 

## 5. Conclusions

The representative panel population developed by shortlisting 117 germplasm lines based on six antioxidant trait phenotypic groups showed wide genetic variation among the germplasm lines. Moreover, the population showed higher diversity parameters based on 131 SSR marker allele data. Therefore, the choice of mapping population was effective for the association mapping study of the six antioxidant traits, *viz.*, catalase, peroxidase, CUPRAC, DPPH, FRAP and TPC, using the population of 117 lines and 131 SSR markers. Donor lines rich in multiple antioxidant traits were identified from the population for antioxidant improvement programs. The population was classified into three genetic groups and showed reasonable correspondence with the antioxidant traits. The presence of a linkage disequilibrium in the population was confirmed from the estimated Fst values. A total of 11 significant marker–trait associations for antioxidant enzymes and activities was detected for three QTLs, namely, *qACD2.1*, *qACD11.1* and *qACD12.2* for DPPH; *qCAT8.1* and *qCAT11.1* for catalase; *qFRAP11.1, qFRAP12.1* and *qFRAP12.2* for FRAP, and *qCUPRAC3.1*, q*CUPRAC11.1* and *qCUPRA12.1* for CUPRAC. Co-localization of the QTLs was detected for *qACD11.1, qFRAC11.1* and q*CUPRAC11.1* regulating DPPH, FRAP and CUPRAC activities, respectively, and *qACD12.2 and qFRAP12.1* remained close on chromosome 12. These QTLs will be useful in antioxidant improvement programs in rice.

## Figures and Tables

**Table 1:** Mean values of catalase (unit min^−1^ g^−1^), peroxidase (unit min^−1^ g^−1^), TPC (mg CE100 g^−1^), DPPH (% inhibition), FRAP (AAE g^−1^) and CUPRAC (TE g^−1^) antioxidant traits estimated from the 117 genotypes present in the panel population.

Sl. No.	Kernel Color	Genotype/Vernacular Name/Accession No.	Catalase	Peroxidase	TPC	DPPH	FRAP	CUPRAC
1	White	AC.5993	0.108	0.0002	14.432	8.149	4.142	25.167
2	White	AC.6221	0.216	0.0003	3.136	19.613	7.313	24.958
3	White	AC.6023	0.130	0.0011	2.295	8.149	6.007	25.833
4	White	AC.6172	0.065	0.0003	9.295	4.420	6.045	27.458
5	White	AC.6027	0.065	0.0005	0.232	11.050	11.978	31.500
6	White	AC.6007	0.173	0.0005	11.250	3.039	4.590	22.875
7	White	AC.9021	0.130	0.0005	34.091	4.144	12.360	24.958
8	Red	AC.9028	0.628	0.0008	205.568	58.011	27.575	49.958
9	White	AC.9030	1.082	0.0007	35.000	11.326	5.821	26.417
10	White	AC.9035	0.152	0.0013	41.364	5.249	8.955	29.958
11	Red	AC.9038	0.065	0.0002	118.523	42.064	31.306	44.542
12	White	AC.9043	0.152	0.0005	31.477	12.155	12.052	25.792
13	White	AC.9044A	0.152	0.0006	23.295	16.713	14.813	23.917
14	Red	AC.20920	0.108	0.0002	134.886	81.107	24.963	117.875
15	Red	AC.20907	0.108	0.0003	148.182	86.645	27.164	118.083
16	White	AC.20845	0.108	0.0008	34.773	22.801	5.075	21.208
17	Red	AC.20770	0.152	0.0005	94.545	84.202	23.694	89.125
18	Red	AC.20627	0.173	0.0003	144.545	82.736	28.470	134.958
19	White	AC.20686	0.065	0.0004	8.636	4.560	4.179	24.125
20	White	AC.20664	0.130	0.0007	45.455	13.192	7.276	25.792
21	Red	AC.20614	0.173	0.0003	148.523	85.342	20.858	101.208
22	White	Jhagrikartik	0.152	0.0002	34.545	8.143	4.739	32.042
23	White	Dadghani	0.195	0.0004	26.136	21.010	5.000	28.083
24	White	Shayam	0.974	0.0001	39.318	18.567	5.299	29.542
25	White	Basumati-B	0.238	0.0001	26.364	22.801	7.500	27.875
26	Red	Bharati	0.195	0.0002	114.545	80.130	20.709	41.292
27	White	Joha	0.173	0.0001	10.000	17.590	5.187	21.625
28	Red	Adira-1	0.130	0.0002	51.250	78.372	32.127	123.292
29	Red	Adira-2	0.130	0.0005	70.000	83.206	35.336	132.667
30	Red	Adira-3	0.108	0.0002	103.864	80.407	31.381	107.875
31	Red	PK6	0.130	0.0005	62.386	84.478	30.448	99.125
32	Red	VAC.haw	0.130	0.0005	84.889	77.075	28.784	58.917
33	Red	Kozhivalan	0.152	0.0006	87.893	72.392	29.463	61.833
34	Red	Marathondi	0.108	0.0007	71.023	78.880	27.440	60.583
35	Red	Ezhoml-2	0.260	0.0003	78.182	88.295	24.142	76.208
36	Red	Jyothi	0.065	0.0005	121.818	87.786	21.716	75.792
37	Red	Kantakapura	0.152	0.0003	113.636	90.228	31.082	118.917
38	Red	Kantakaamala	0.152	0.0006	128.750	90.554	28.619	109.958
39	Red	Kapanthi	0.108	0.0004	131.705	89.902	24.515	43.074
40	White	Karpurkanti	0.108	0.0004	45.909	29.967	6.866	31.333
41	Red	Kathidhan	0.173	0.0004	148.295	89.577	22.836	76.208
42	Red	Kundadhan	0.130	0.0001	160.909	89.902	31.642	59.542
43	Red	Champaeisiali	0.108	0.0003	136.818	87.296	23.821	77.875
44	White	Latamahu	0.260	0.0007	33.295	24.202	14.104	11.875
45	Red	Latachaunri	0.238	0.0015	128.068	91.531	24.634	48.292
46	White	AC.10608	0.087	0.0008	1.182	14.503	3.769	21.208
47	White	AC.10187	0.087	0.0004	31.477	34.586	10.261	35.792
48	Red	AC.10162	0.065	0.0007	83.409	76.796	24.888	64.333
49	White	AC.7282	0.108	0.0008	1.477	9.807	5.784	22.250
50	White	AC.7269	0.108	0.0004	1.722	12.569	5.709	23.500
51	White	AC.7134	0.087	0.0007	3.636	12.431	5.746	31.208
52	White	AC.7008	0.108	0.0005	1.864	23.260	9.701	32.458
53	White	AC.9093	0.714	0.0004	8.750	24.309	7.201	20.792
54	White	AC.9090	0.173	0.0006	26.591	22.238	6.866	19.958
55	White	AC.9076A	0.152	0.0005	21.250	9.945	6.418	20.792
56	Red	AC.9065	0.390	0.0004	126.591	42.486	26.604	56.417
57	Red	AC.9063	0.108	0.0007	152.500	31.878	26.007	61.208
58	White	AC.9058	0.238	0.0003	36.591	15.193	9.739	16.833
59	White	AC.9053A	0.195	0.0010	23.523	12.017	9.515	28.708
60	Red	AC.9050	0.411	0.0011	116.477	38.370	23.657	56.833
61	White	AC.9005	0.152	0.0003	13.409	1.657	5.784	24.542
62	White	AC.20389	0.108	0.0004	1.409	18.730	3.769	23.917
63	White	AC.20371	0.108	0.0005	19.773	28.664	10.187	24.958
64	Red	AC.20423	0.130	0.0007	141.591	59.772	23.396	71.208
65	White	AC.20362	0.152	0.0007	11.705	21.661	10.112	31.625
66	White	AC.20328	0.152	0.0007	20.909	23.290	7.910	48.208
67	White	AC.20317	0.065	0.0003	21.250	28.990	11.157	37.667
68	Red	AC.20282	0.152	0.0003	169.091	87.948	42.127	202.250
69	Red	AC.20246	0.238	0.0002	165.227	86.971	39.366	187.875
70	Red	AC.20347	0.065	0.0003	132.727	36.156	28.060	47.250
71	White	Palinadhan-1	0.065	0.0004	27.386	19.577	5.448	21.208
72	White	Chatuimuchi	0.108	0.0003	25.795	21.824	5.037	26.625
73	White	Uttarbangalocal-9	0.216	0.0006	11.364	20.521	4.776	27.875
74	White	Gochi	0.216	0.0004	5.227	19.479	11.381	21.208
75	White	Sugandha-2	0.152	0.0002	16.932	27.850	6.679	26.000
76	White	Jhingesal	0.801	0.0001	11.136	14.658	4.813	36.208
77	Red	Cheruvirippu	0.195	0.0002	46.932	88.804	22.425	73.917
78	Red	Mahamaga	0.065	0.0007	111.750	77.608	31.903	55.792
79	White	Jaya	0.065	0.0005	1.705	24.427	10.299	34.958
80	Red	D1	0.152	0.0006	78.864	79.898	30.261	53.292
81	Red	Pk-21	0.152	0.0007	62.841	88.041	25.522	59.542
82	White	Gandhakasala	0.152	0.0004	2.727	24.427	9.739	28.083
83	Red	Sreyas	0.108	0.0007	117.500	84.860	26.604	91.208
84	Red	GondiAC.hampeisiali	0.238	0.0005	122.273	91.694	27.612	42.250
85	White	Chinamal	0.195	0.0004	28.864	23.779	11.567	23.292
86	White	Magra	0.173	0.0004	21.477	23.453	16.604	24.750
87	Red	Landi	0.087	0.0004	104.659	89.577	25.075	38.292
88	White	Lalgundi	0.238	0.0003	33.182	26.384	9.291	18.708
89	White	BalisaralaktimAC.hi	0.346	0.0002	14.773	12.378	13.955	27.250
90	White	Laxmibilash	0.541	0.0002	17.727	23.127	9.216	16.625
91	Red	Kaniar	0.390	0.0001	82.955	89.251	26.418	48.083
92	White	Kanakchampa	0.368	0.0004	17.273	18.567	6.903	2.833
93	White	Magura-s	0.303	0.0002	17.273	5.700	10.560	3.542
94	White	AC.44603	0.216	0.0003	32.273	34.351	11.940	36.208
95	Red	AC.44585	0.346	0.0009	85.227	48.295	29.963	48.917
96	White	AC.44598	0.152	0.0002	20.000	18.321	8.955	24.542
97	Red	AC.44592	0.216	0.0006	68.068	83.969	34.104	151.833
98	Red	AC.44646	0.866	0.0004	65.000	83.461	44.813	211.625
99	White	AC.44604	0.390	0.0003	19.886	12.276	12.201	35.375
100	White	AC.44597	0.108	0.0004	32.955	38.168	16.567	16.208
101	White	AC.44638	0.173	0.0003	25.795	15.776	10.672	27.250
102	Red	AC.44595	0.519	0.0003	54.659	84.478	49.440	238.708
103	Red	AC.44588	0.346	0.0002	54.545	83.715	35.149	186.417
104	Red	AC.44591	0.087	0.0004	94.773	46.387	35.597	57.458
105	Red	AC.44594	0.390	0.0002	62.273	86.005	29.507	114.750
106	Red	AC.43737	0.411	0.0003	52.500	88.931	36.642	166.000
107	White	AC.43660	0.563	0.0003	44.432	26.005	11.231	47.875
108	White	AC.43732	0.108	0.0002	41.250	26.768	25.672	59.542
109	White	AC.43661	0.238	0.0003	36.818	34.020	14.627	39.542
110	Red	AC.43738	0.606	0.0002	75.341	86.768	40.933	174.958
111	White	AC.43669	0.238	0.0002	45.000	27.405	15.746	18.083
112	White	AC.43663	0.238	0.0003	22.045	25.496	9.478	24.750
113	Red	AC.43658	0.130	0.0004	86.364	85.496	38.843	93.708
114	White	AC.43662	0.130	0.0004	21.250	23.282	10.634	34.333
115	Red	AC.43670	0.238	0.0004	80.455	88.550	59.142	287.042
116	White	AC.43675	0.303	0.0003	48.636	16.768	11.716	39.750
117	Red	AC.43676	0.281	0.0003	45.341	73.664	35.522	149.125
Mean			0.219	0.000	58.365	44.918	18.435	58.691
CV %			10.75	11.32	5.78	4.64	4.72	5.81
LSD_5%_			0.0448	0.00009	6.122	3.303	1.579	6.54

CAT: catalase (unit min ^−1^ g^−1^); PEROX: peroxidase (unit min ^−1^ g^−1^); TPC: Total phenolics content (mg catechol or CE 100 g^−1^); DPPH: 2,2-diphenyl-1-picrylhydrazyl (% inhibition); FRAP: Ferric reducing antioxidant power Activity (µg ascorbic acid equivalent or AAE) g^−1^; CUPRAC: Cupric ion reducing antioxidant capacity (μg trolox equivalent or TE) g^−1^).

**Table 2:** The inferred ancestry value and population structure of individual members in the panel population with their antioxidant trait classification.

Sl. No.	Accession No./ Vernacular Name of Germplasm Line	Inferred Ancestry Value at K = 3			Group	Antioxidants Traits in Each Germplasm Line
		Q1	Q2	Q3		
1	AC5993	0.995	0.003	0.003	SP1	Very low
2	AC6170	0.992	0.006	0.002	SP1	Low
3	AC6023	0.981	0.017	0.002	SP1	High Peroxidase
4	AC6172	0.961	0.037	0.002	SP1	Low
5	AC6027	0.009	0.002	0.989	SP3	Very low
6	AC9006	0.995	0.003	0.002	SP1	Very low
7	AC9021	0.986	0.011	0.003	SP1	Very low
8	AC9028	0.937	0.06	0.003	SP1	High Peroxidase
9	AC9030	0.994	0.005	0.001	SP1	High Catalase
10	AC9035	0.987	0.004	0.009	SP1	High Peroxidase
11	AC9038	0.998	0.001	0.001	SP1	Very low
12	AC9043	0.998	0.001	0.001	SP1	Low
13	AC9044	0.994	0.002	0.003	SP1	Very low
14	AC20920	0.995	0.002	0.003	SP1	High DPPH
15	AC20907	0.997	0.002	0.001	SP1	High DPPH
16	AC20845	0.997	0.002	0.001	SP1	High Peroxidase
17	AC20770	0.997	0.001	0.002	SP1	High DPPH
18	AC20627	0.998	0.001	0.001	SP1	High DPPH
19	AC20686	0.997	0.002	0.001	SP1	Very low
20	AC20664	0.996	0.002	0.001	SP1	Low
21	AC20614	0.996	0.001	0.003	SP1	High DPPH
22	Jhagrikarti	0.99	0.009	0.002	SP1	Very low
23	Dadghani	0.991	0.005	0.004	SP1	Very low
24	Shayam	0.004	0.002	0.994	SP3	High Catalase
25	Basumati	0.138	0.004	0.859	SP3	Low
26	Bharati	0.997	0.001	0.002	SP1	High DPPH
27	Joha	0.997	0.001	0.002	SP1	Very low
28	Adira-1	0.613	0.018	0.369	A	Medium
29	Adira-2	0.997	0.002	0.001	SP1	High DPPH
30	Adira-3	0.622	0.003	0.376	A	High DPPH
31	PK6	0.969	0.003	0.028	SP1	High DPPH
32	Vachaw	0.946	0.002	0.053	SP1	High DPPH
33	Kozhivalan	0.998	0.002	0.001	SP1	High DPPH
34	Marathondi	0.515	0.022	0.463	A	High DPPH
35	Ezhoml-2	0.998	0.001	0.001	SP1	High DPPH
36	Jyothi	0.998	0.001	0.001	SP1	High DPPH
37	Kantakopura	0.997	0.001	0.002	SP1	High DPPH
38	Kantakaamal	0.693	0.081	0.227	A	High DPPH
39	Kapanthi	0.302	0.323	0.375	A	High DPPH
40	Karpurkanti	0.002	0.001	0.997	SP3	Very low
41	Kathidhan	0.899	0.095	0.006	SP1	High DPPH
42	Kundadhan	0.995	0.004	0.001	SP1	High TPC and DPPH
43	Champaeisia	0.991	0.003	0.006	SP1	High DPPH
44	Latamahu	0.996	0.002	0.002	SP1	Low
45	Latachaunri	0.994	0.004	0.002	SP1	High DPPH and Peroxidase
46	AC10608	0.995	0.005	0.001	SP1	High Peroxidase
47	AC10187	0.945	0.052	0.002	SP1	High DPPH
48	AC10162	0.963	0.021	0.016	SP1	High DPPH
49	AC7282	0.002	0.001	0.997	SP1	High Peroxidase
50	AC7269	0.997	0.002	0.001	SP1	Very low
51	AC7134	0.785	0.006	0.208	A	Low
52	AC7008	0.998	0.001	0.001	SP1	Low
53	AC9093	0.996	0.001	0.003	SP1	High Catalase
54	AC9090	0.993	0.003	0.004	SP1	Very low
55	AC9076A	0.995	0.003	0.001	SP1	Low
56	AC9065	0.973	0.003	0.024	SP1	Low
57	AC9063	0.993	0.006	0.001	SP1	High TPC
58	AC9058	0.998	0.001	0.001	SP1	Low
59	AC9053A	0.831	0.158	0.011	SP1	High Peroxidase
60	AC9050	0.994	0.001	0.004	SP1	Low
61	AC9005	0.994	0.004	0.002	SP1	Low
62	AC20389	0.945	0.035	0.02	SP1	Low
63	AC20371	0.993	0.006	0.001	SP1	Low
64	AC20423	0.996	0.003	0.001	SP1	Low
65	AC20362	0.977	0.018	0.005	SP1	Low
66	AC20328	0.986	0.008	0.006	SP1	Low
67	AC20317	0.996	0.002	0.002	SP1	Low
68	AC20282	0.889	0.104	0.007	SP1	High CUPRAC, Cata, TPC, DPPH and FRAP
69	AC20246	0.894	0.017	0.089	SP1	High CUPRAC, FRAP and DPPH
70	AC20347	0.943	0.055	0.002	SP1	Low
71	Palinadhan-	0.334	0.214	0.452	A	High DPPH
72	Chatuimuchi	0.001	0.001	0.998	SP3	Very low
73	Uttarbangal	0.904	0.094	0.002	SP1	Very low
74	Gochi	0.941	0.053	0.006	SP1	High DPPH
75	Sugandha-2	0.002	0.001	0.998	SP3	Very low
76	Jhingesal	0.997	0.002	0.001	SP1	High Catatalase
77	Cheruviripp	0.996	0.003	0.001	SP1	High DPPH
78	Mahamaga	0.985	0.013	0.002	SP1	High DPPH
79	Jaya	0.991	0.008	0.001	SP1	Low
80	D1	0.944	0.033	0.023	SP1	High DPPH
81	PK21	0.985	0.014	0.001	SP1	High DPPH
82	Gandhakasal	0.004	0.003	0.993	SP3	Very low
83	Sreyas	0.995	0.002	0.003	SP1	High DPPH
84	Gondiachamp	0.995	0.002	0.003	SP1	High DPPH
85	Chinamal	0.981	0.001	0.017	SP1	Low
86	Magra	0.995	0.002	0.003	SP1	Very low
87	Landi	0.997	0.001	0.002	SP1	High DPPH
88	Lalgundi	0.99	0.003	0.007	SP1	Very low
89	Balisaralak	0.994	0.003	0.003	SP1	Very low
90	Laxmibilash	0.426	0.002	0.572	A	Low
91	Kaniar	0.98	0.005	0.016	SP1	High DPPH
92	Kanakchampa	0.976	0.003	0.02	SP1	Very low
93	Magura-S	0.895	0.001	0.104	SP1	Very low
94	AC44603	0.017	0.981	0.001	SP2	Low
95	AC44585	0.004	0.984	0.012	SP2	High Peroxidase
96	AC44598	0.007	0.987	0.006	SP2	Low
97	AC44592	0.995	0.003	0.003	SP2	High DPPH
98	AC44646	0.001	0.997	0.001	SP2	High Cata, DPPH, FRAP and CUPRAC
99	AC44604	0.001	0.998	0.001	SP2	Medium
100	AC44597	0.013	0.98	0.007	SP2	Medium
101	AC44638	0.002	0.997	0.001	A	Low
102	AC44595	0.001	0.284	0.715	SP2	High CUPRAC, FRAP and DPPH
103	AC44588	0.005	0.994	0.002	SP2	High CUPRAC, DPPH and FRAP
104	AC44591	0.002	0.997	0.001	SP2	Low
105	AC44594	0.002	0.998	0.001	SP2	High DPPH
106	AC43737	0.01	0.988	0.002	SP2	High DPPH and CUPRAC
107	AC43660	0.002	0.997	0.001	SP2	High Catalase, DPPH, FRAP, CUPRAC
108	AC43732	0.003	0.996	0.001	SP2	High Catalase, DPPH and CUPRAC
109	AC43661	0.001	0.998	0.001	SP2	Low
110	AC43738	0.004	0.995	0.001	SP2	High Catalase and CUPRAC
111	AC43669	0.002	0.997	0.001	SP2	High DPPH
112	AC43663	0.003	0.994	0.003	SP2	High DPPH
113	AC43658	0.001	0.997	0.002	SP2	High DPPH
114	AC43662	0.001	0.998	0.001	SP2	Low
115	AC43670	0.003	0.981	0.016	SP2	High DPPH and CUPRAC
116	AC43675	0.002	0.805	0.193	SP2	High DPPH
117	AC43676	0.002	0.986	0.012	SP2	Medium

**Table 3:** Analysis of molecular variance (AMOVA) of the subpopulations of the panel population for antioxidant properties in 117 rice genotypes.

Source of Variation	AMOVA for the Four Subpopulations at K = 3			
	df.	Mean Sum of Squares	Variance Components	Percentage Variation
Among populations	3	0.641	0.003	1%
Among individuals (accessions) within population	113	0.514	0.022	4%
Within individuals (accessions)	117	0.470	0.470	95%
Total	233		0.495	
F-Statistics	Value	*p*-value		
F_ST_	0.006	0.121		
F_IS_	0.045	0.003		
F_IT_	0.051	0.001		
F_ST_ max.		0.014		
F’_ST_		0.460		

**Table 4:** Marker–trait associations with antioxidant traits catalase, peroxidase, DPPH, FRAP and CUPRAC in the panel population detected by both GLM and MLM at *p* < 0.01.

Sl. No.	Antioxidant Enzymes	Marker	Position (cM)	GLM				MLM			
				Marker_F	Marker_*p*	Marker_R^2^	*q*-Value	Marker_F	Marker_*p*	Marker_R^2^	*q*-Value
1	Catalase	RM1341	80.2	9.99747	0.00204	0.08016	0.00564	7.8566	0.006	0.07179	0.00994
2	Catalase	RM3231	32.7	10.55577	0.00154	0.08424	0.00564	8.16013	0.00514	0.07457	0.009638
3	DPPH	RM247	32.3	10.72025	0.00142	0.08855	0.00966	10.07384	0.00196	0.09162	0.006125
4	DPPH	RM3701	45.3	11.90813	7.99 × 10^−4^	0.09738	0.00564	11.09733	0.00118	0.10093	0.006125
5	DPPH	RM13600	110.2	9.40651	0.00273	0.0779	0.00652	6.88723	0.00994	0.06264	0.00994
6	FRAP	RM247	32.2	9.40651	0.00273	0.0779	0.00617	7.14597	0.00868	0.06551	0.00994
7	FRAP	RM3701	45.3	9.11231	0.00317	0.06781	0.00617	8.98419	0.00338	0.08236	0.007243
8	FRAP	RM309	74.5	12.35495	6.44 × 10^−4^	0.08946	0.00617	7.35763	0.00777	0.06745	0.00994
9	CUPRAC	RM3701	45.3	14.56812	2.26 × 10^−4^	0.10344	0.00771	9.65365	0.00241	0.08678	0.006125
10	CUPRAC	RM235	101.8	9.11231	0.00317	0.06781	0.00564	10.03931	0.00199	0.09024	0.006125
11	CUPRAC	RM148	142.3	12.35495	6.44 × 10^−4^	0.08946	0.00966	7.03523	0.0092	0.06324	0.00994

## Data Availability

The data generated or analyzed in this study are included in this article.

## Supplementary Materials

The following supporting information can be downloaded at: https://www.mdpi.com/article/10.3390/plants11111463/s1, Supplementary Figure S1. Scree plot and loadings generated by the six antioxidant traits and eigen values % in the 117 rice germplasm lines. Supplementary Figure S2. (A) Graph of ∆K value, to the rate of change in the log probability of data between successive K values; (B) population structure of the 117 germplasm lines of the panel population based on membership probability fractions of individual genotypes at K = 2. Supplementary Figure S3. The distribution pattern of alpha and F_ST_ values: (A) alpha value of the population at K = 3 and (B) four subpopulations at K = 3 showing a symmetric shape of the 3 Fst values. Supplementary Table S1. Mean values of catalase, peroxidase, TPC, DPPH, FRAP and CUPRAC antioxidant traits in 270 initial shortlisted genotypes. Supplementary Table S2. Estimation of genetic diversity parameters based on 131 SSR marker loci in a panel containing 117 rice germplasm lines. Supplementary Table S3. The inferred ancestry value and population structure of individual members with their antioxidant classification in the panel population at K = 2. Supplementary Table S4. Marker–trait associations with antioxidant content in the panel population detected by the model GLM at *p* < 0.01. Supplementary Table S5. Marker–trait associations with antioxidants, catalase, peroxidase, TPC, DPPH, FRAP and CUPRAC in the panel population detected by the model MLM at *p* < 0.01.

## Abbreviations

ANOVA	Analysis of variance
CAT	Catalase
CUPRAC	Cupric ion reducing antioxidant capacity
DPPH	2,2-diphenyl-1-picrylhydrazyl
FDR	False discovery rate
FRAP	Ferric reducing antioxidant power
PEROX	Guaicol peroxidase
PIC	Polymorphic information content
RBD	Randomized block design
TPC	Total phenolics content
