# Supplementary material for: Detection of Genomic Regions Controlling the Antioxidant Enzymes, Phenolic Content, and Antioxidant Activities in Rice Grain through Association Mapping
Source: Plants (Basel). 2022 May 30;11(11):1463. doi: 10.3390/plants11111463 (PMC9183076; doi:10.3390/plants11111463)
Supplement: Supplementary file 1 [file plants-11-01463-s001.zip › plants-1733110-supplementary.pdf]

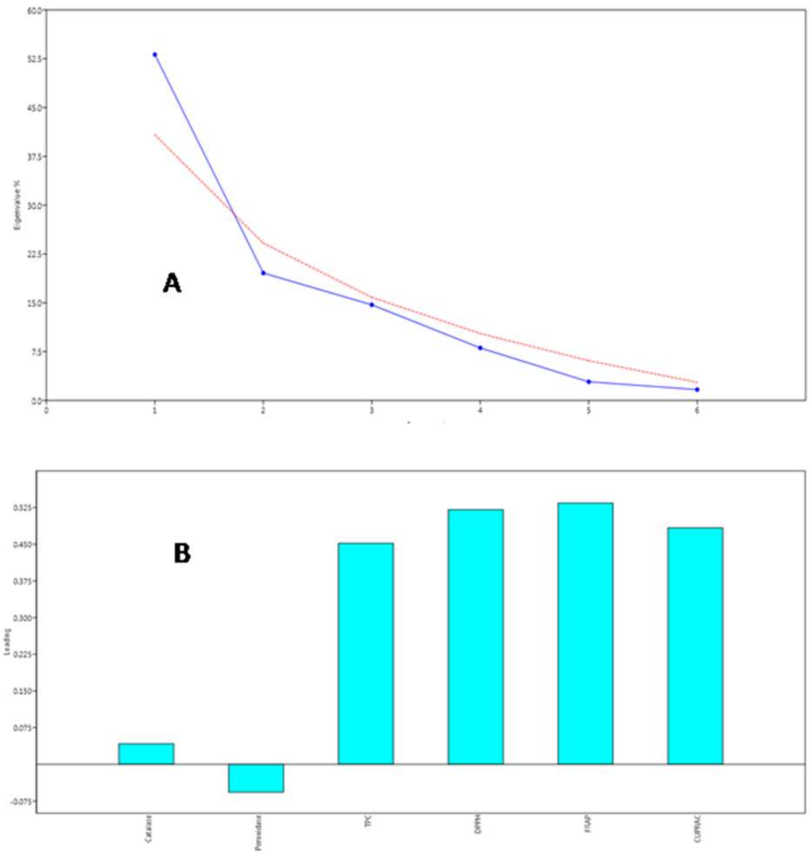

Supplementary Fig. S1.Scree plot annd loadings generated by the six antioxidant traits and eigen values % in the 117 rice germplasm lines.

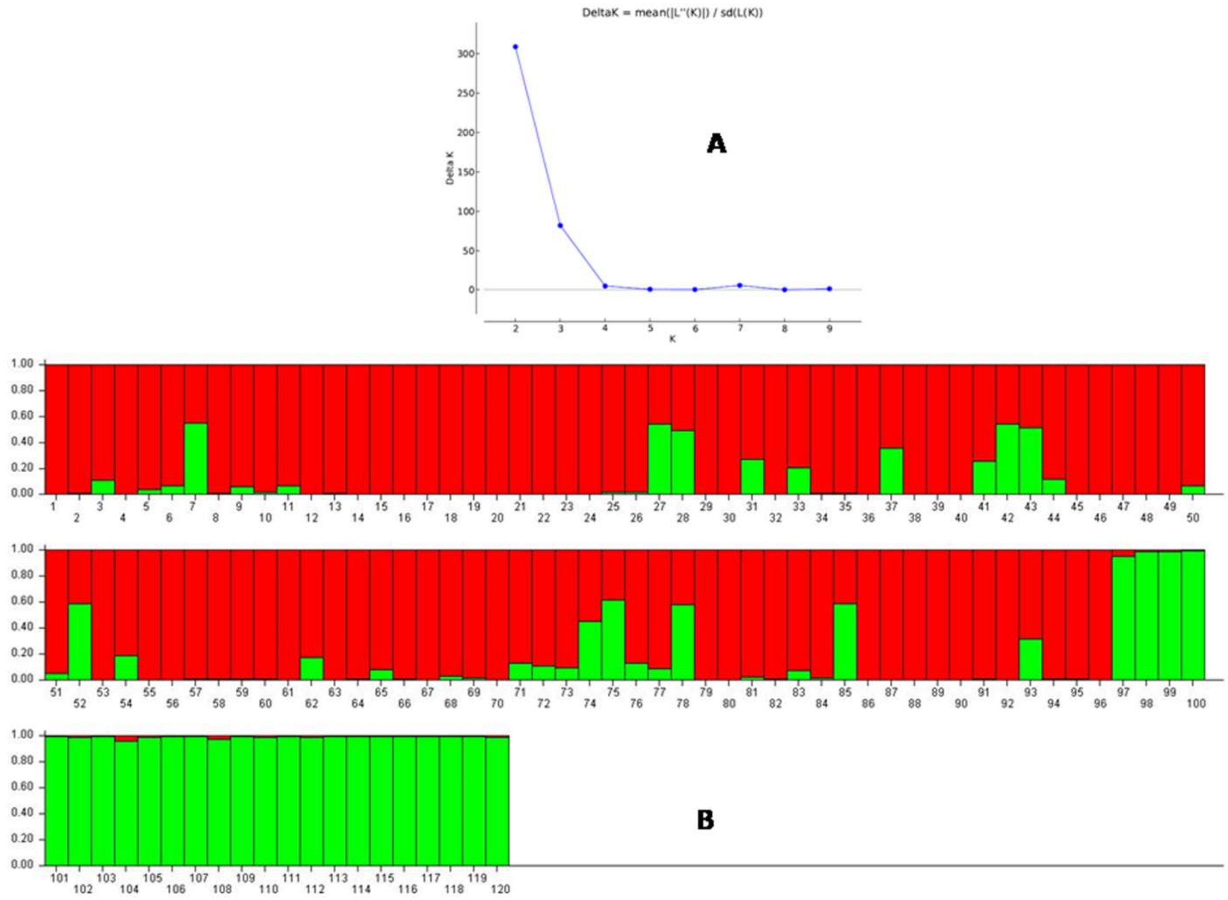

Supplementary Fig. S2. **A)** Graph of Δ K value, to the rate of change in the log probability of data between successive K values; **B)** Population structure of the 117 germplasm lines of the panel population based on membership probability fractions of individual genotypes at K = 2.

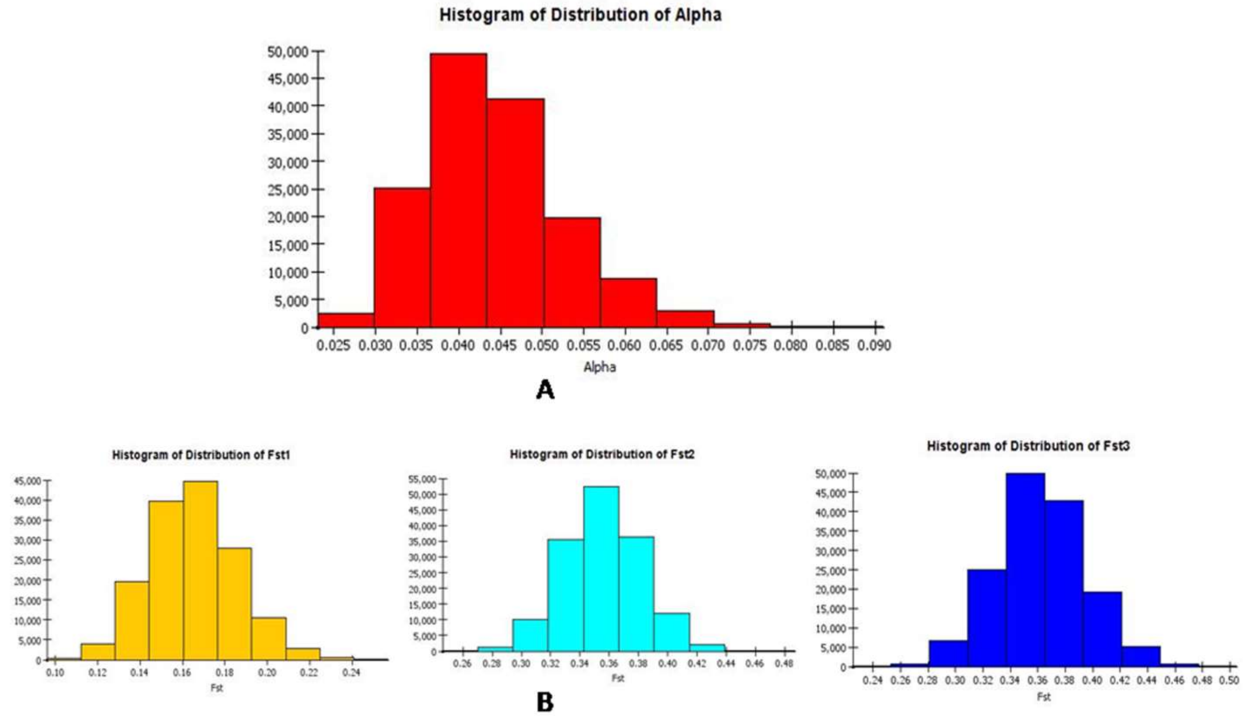

Supplementary Fig. S3. The distribution pattern of alpha and F_{ST} values (A) Alpha value of the population at K = 3 and (B) Four sub-populations at K = 3 showing a symmetric shape of the 3 Fst values.

Supplementary Table S1. Mean vaalues of Mean values of catalase (unit min^{-1} g^{-1}), pperoxidise (unit min^{-1} g^{-1}), TPC (mg CE100 g^{-1}), DPPPH (% inhibition), FRAP (AAE g^{-1}) and CUPRACC (TE g^{-1}) antioxidants traits in 270 initial sshortlisted genotypes

Sl No.	Kernel colour	Genotype accession no./Vernacular name/ Cultivar	CAT	PEROX	TPC	DPPH	FRAP	CUPRAC
1	White	AC9005	0.152	0.000	13.409	1.657	5.784	24.542
2	White	AC9006	0.173	0.001	11.250	3.039	4.590	22.875
3	White	AC9021	0.130	0.001	34.091	4.144	12.360	24.958
4	White	AC9030	1.082	0.001	35.000	11.326	5.821	26.417
5	White	AC9035	0.152	0.001	41.364	5.249	8.955	29.958
6	White	AC9043	0.152	0.000	31.477	12.155	12.052	25.792
7	White	AC9044A	0.152	0.001	23.295	16.713	14.813	23.917
8	White	AC9053A	0.195	0.001	23.523	12.017	9.515	28.708
9	White	AC9058	0.238	0.000	36.591	15.193	9.739	16.833
10	White	AC9076A	0.152	0.001	21.250	9.945	6.418	20.792
11	White	AC9090	0.173	0.001	26.591	22.238	6.866	19.958
12	White	AC9093	0.714	0.000	8.750	24.309	7.201	20.792
13	White	AC9102	0.173	0.000	18.636	28.000	8.130	24.665

14	White	AC9119A	0.098	0.001	21.591	26.800	10.570	24.705
15	White	AC20317	0.065	0.000	21.250	28.990	11.157	37.667
16	White	AC20328	0.152	0.001	20.909	23.290	7.910	48.208
17	White	AC20362	0.152	0.001	11.705	21.661	10.112	31.625
18	White	AC 20371	0.108	0.001	19.773	28.664	10.187	24.958
19	White	AC 20389	0.108	0.000	1.409	18.730	3.769	23.917
20	White	AC20664	0.130	0.001	45.455	13.192	7.276	25.792
21	White	AC20686	0.065	0.000	8.636	4.560	4.179	24.125
22	White	AC20845	0.108	0.001	34.773	22.801	5.075	21.208
23	White	AC20436	0.108	0.000	12.386	14.780	10.715	20.850
24	White	AC20604	0.058	0.001	16.818	26.755	14.340	23.660
25	White	AC20690	0.058	0.000	48.864	24.240	10.800	28.635
26	white	Karpurkanti	0.108	0.000	45.909	29.967	6.866	31.333
27	White	Lata mahu	0.260	0.001	33.295	24.202	14.104	11.875
28	White	Chinamal	0.195	0.000	28.864	23.779	11.567	23.292
29	White	Magra	0.173	0.000	21.477	23.453	16.604	24.750
30	White	Lalgundi	0.238	0.000	33.182	26.384	9.291	18.708
31	White	Balisara lakti machi	0.346	0.000	14.773	12.378	13.955	27.250
32	White	Laxmi bilash	0.541	0.000	17.727	23.127	9.216	16.625
33	White	Kanak champa	0.368	0.000	17.273	18.567	6.903	2.833
34	White	Magura-s	0.303	0.000	17.273	5.700	10.560	3.542
35	White	Maguramanji	1.212	0.000	22.045	10.300	11.710	18.450
36	white	Jhagri kartik	0.152	0.000	34.545	8.143	4.739	32.042
37	white	Dad ghani	0.195	0.000	26.136	21.010	5.000	28.083
38	White	Shayam	0.974	0.000	39.318	18.567	5.299	29.542
39	white	Basumati-B	0.238	0.000	26.364	22.801	7.500	27.875
40	White	Joha	0.173	0.000	10.000	17.590	5.187	21.625
41	White	Jhingesal	0.801	0.000	11.136	14.658	4.813	36.208
42	White	Sugandha-2	0.152	0.000	16.932	27.850	6.679	26.000
43	White	Gochi	0.216	0.000	5.227	19.479	11.381	21.208
44	White	Uttarbanga local-9	0.216	0.001	11.364	20.521	4.776	27.875
45	White	Chatui muchi	0.108	0.000	25.795	21.824	5.037	26.625
46	White	Palina dhan-1	0.065	0.000	27.386	19.577	5.448	21.208
47	White	Jaya	0.065	0.001	1.705	24.427	10.299	34.958
48	White	Gandhakasala	0.152	0.000	2.727	24.427	9.739	28.083
49	White	Gerwathor	0.098	0.000	11.234	18.525	6.339	12.353
50	White	Kaikee	0.105	0.000	12.841	21.260	10.890	19.665
51	White	Manahar	0.098	0.000	13.068	13.707	12.760	19.960

		rathori						
52	White	Bakuldhan	0.152	0.000	15.682	26.307	8.631	14.595
53	White	AC-5993	0.108	0.000	14.432	8.149	4.142	25.167
54	White	AC-6007	0.108	0.000	1.523	13.260	5.672	23.958
55	White	AC-6023	0.130	0.001	2.295	8.149	6.007	25.833
56	White	AC-6027	0.065	0.001	0.232	11.050	11.978	31.500
57	White	AC-6170	0.216	0.000	3.136	19.613	7.313	24.958
58	White	AC-6172	0.065	0.000	9.295	4.420	6.045	27.458
59	white	AC-6183	0.065	0.001	15.000	12.707	6.866	31.000
60	White	AC-6221	0.108	0.000	1.295	5.249	7.276	26.417
61	white	AC-7008	0.108	0.000	1.864	23.260	9.701	32.458
62	White	AC-7134	0.087	0.001	3.636	12.431	5.746	31.208
63	White	AC-7269	0.108	0.000	1.722	12.569	5.709	23.500
64	White	AC-7282	0.108	0.001	1.477	9.807	5.784	22.250
65	White	AC-10187	0.087	0.000	31.477	34.586	10.261	35.792
66	White	AC-10608	0.087	0.001	1.182	14.503	3.769	21.208
67	White	AC-6006	0.173	0.000	3.523	18.305	11.145	25.006
68	White	AC-6617	0.108	0.000	11.704	15.655	12.175	12.625
69	White	AC-7009	0.087	0.000	3.864	6.300	7.265	12.765
70	White	AC-7124	0.123	0.000	13.284	20.718	13.310	28.360
71	White	AC-7204	0.055	0.001	1.705	16.400	10.315	21.295
72	White	AC-10333	0.045	0.000	1.705	13.165	7.867	24.614
73	White	AC-10438	0.195	0.000	3.523	14.300	6.290	18.795
74	White	AC- 44597	0.108	0.000	32.955	38.168	16.567	16.208
75	White	AC- 44598	0.152	0.000	20.000	18.321	8.955	24.542
76	White	AC- 44638	0.173	0.000	25.795	15.776	10.672	27.250
77	White	AC- 44603	0.216	0.000	32.273	34.351	11.940	36.208
78	White	AC- 44604	0.390	0.000	19.886	12.276	12.201	35.375
79	White	AC-44319	0.519	0.002	30.568	24.224	12.537	16.250
80	White	AC-44586	0.260	0.001	51.364	25.369	13.993	13.417
81	White	AC-44602	0.411	0.000	17.386	19.084	10.448	26.833
82	White	AC-44601	0.095	0.000	23.068	18.039	12.799	20.292
83	White	AC-44608	0.173	0.000	38.182	18.575	12.500	28.292
84	White	AC-44613	0.346	0.000	27.159	22.901	12.201	24.333
85	White	AC-44649	0.152	0.000	28.182	23.155	12.276	18.292
86	White	AC43660	0.563	0.000	44.432	26.005	11.231	47.875
87	White	AC43661	0.238	0.000	36.818	34.020	14.627	39.542
88	White	AC43662	0.130	0.000	21.250	23.282	10.634	34.333
89	White	AC43663	0.238	0.000	22.045	25.496	9.478	24.750

90	White	AC 43669	0.238	0.000	45.000	27.405	15.746	18.083
91	White	AC43732	0.108	0.000	41.250	26.768	25.672	59.542
92	White	AC43675	0.303	0.000	48.636	16.768	11.716	39.750
93	White	Kalinga-2	0.129	0.000	40.057	23.201	18.825	19.190
94	White	Kalinga-3	0.107	0.000	47.443	16.367	23.020	20.200
95	White	Hazaridhan	0.132	0.000	28.896	19.424	20.675	19.565
96	White	Satabdi	0.152	0.000	36.648	43.165	17.705	20.200
97	White	Swarna sub-1	0.160	0.000	28.693	34.173	12.365	19.435
98	White	Naveen	0.168	0.000	35.795	41.187	15.695	20.405
99	White	Satyabhama	0.241	0.000	37.898	47.842	16.150	21.995
100	White	Gitanjali	0.113	0.001	20.705	17.230	17.525	22.985
101	White	CR Dhan 305	0.155	0.000	35.966	25.899	20.190	23.430
102	White	CR Dhan 310	0.143	0.000	27.045	11.871	21.687	20.995
103	White	CR Dhan 907	0.220	0.000	39.205	22.662	20.485	20.625
104	White	Govindbhog	0.259	0.001	26.943	7.734	21.202	21.500
105	White	Nuakalazeera	0.282	0.001	32.386	13.849	18.845	19.930
106	White	Masuri	0.100	0.000	32.216	26.871	19.850	19.090
107	White	Heera	0.141	0.000	24.830	18.345	20.410	19.770
108	White	Nuadhusara	0.160	0.000	39.489	19.604	19.550	17.750
109	White	Banskathi	0.147	0.000	35.227	11.331	21.580	20.300
110	White	Hanseswari	0.102	0.000	24.489	26.691	22.625	21.440
111	White	Savitri	0.125	0.001	31.989	13.849	23.541	22.685
112	White	Dhalaheera	0.125	0.000	31.932	12.698	22.628	20.505
113	White	Mahulata	0.139	0.000	22.045	37.050	20.212	17.470
114	White	Padmini	0.168	0.001	27.818	10.072	21.298	15.820
115	White	Ratna	0.177	0.000	26.534	36.691	16.190	17.240
116	White	Annada	0.237	0.000	22.273	25.540	17.015	20.400
117	White	Gomati	0.090	0.000	27.159	12.338	18.280	22.200
118	White	Lalat	0.170	0.001	30.966	22.662	19.905	20.740
119	White	Chinikamini	0.158	0.000	10.705	26.877	20.210	20.815
120	White	CR Dhan 801	0.073	0.000	18.983	16.105	21.925	22.105
121	White	Pratikshya	0.124	0.000	28.505	27.370	20.388	21.610
122	Red	Landi	0.087	0.000	104.659	89.577	25.075	38.292
123	Red	AC9050	0.411	0.001	116.477	38.370	23.657	56.833
124	Red	AC9038	0.065	0.000	118.523	42.064	31.306	44.542
125	Red	AC9028	0.628	0.001	205.568	58.011	27.575	49.958
126	Red	AC9063	0.108	0.001	152.500	31.878	26.007	61.208
127	Red	AC9065	0.390	0.000	126.591	42.486	26.604	56.417
128	Red	AC9011	0.130	0.000	148.295	41.260	22.910	42.725

129	Red	AC20246	0.238	0.000	165.227	86.971	39.366	187.875
130	Red	AC20282	0.152	0.000	169.091	87.948	42.127	202.250
131	Red	AC 20423	0.130	0.001	141.591	59.772	23.396	71.208
132	Red	AC 20614	0.173	0.000	148.523	85.342	20.858	101.208
133	Red	AC 20627	0.173	0.000	144.545	82.736	28.470	134.958
134	Red	AC20770	0.152	0.001	94.545	84.202	23.694	89.125
135	Red	AC20907	0.108	0.000	148.182	86.645	27.164	118.083
136	Red	AC20920	0.108	0.000	134.886	81.107	24.963	117.875
137	Red	AC 20347	0.065	0.000	132.727	36.156	28.060	47.250
138	Red	Kanta kapura	0.152	0.000	113.636	90.228	31.082	118.917
139	Red	Kantakaamala	0.152	0.001	128.750	90.554	28.619	109.958
140	Red	Kapanthi	0.108	0.000	131.705	89.902	24.515	43.074
141	Red	Kathidhan	0.173	0.000	148.295	89.577	22.836	76.208
142	Red	Kunda dhan	0.130	0.000	160.909	89.902	31.642	59.542
143	Red	Champaeisiali	0.108	0.000	136.818	87.296	23.821	77.875
144	Red	Lata chaunri	0.238	0.002	128.068	91.531	24.634	48.292
145	Red	Gondia champeisiali	0.238	0.001	122.273	91.694	27.612	42.250
146	Red	Kaniar	0.390	0.000	82.955	89.251	26.418	48.083
147	Red	Adira-1	0.130	0.000	51.250	78.372	32.127	123.292
148	Red	Adira-2	0.130	0.001	70.000	83.206	35.336	132.667
149	Red	Adira-3	0.108	0.000	103.864	80.407	31.381	107.875
150	Red	PK6	0.130	0.001	62.386	84.478	30.448	99.125
151	Red	Vachaw	0.130	0.000	84.889	77.075	28.784	58.917
152	Red	Kozhivalan	0.152	0.001	87.893	72.392	29.463	61.833
153	Red	Marathondi	0.108	0.001	71.023	78.880	27.440	60.583
154	Red	Ezhoml-2	0.260	0.000	78.182	88.295	24.142	76.208
155	Red	Cheruvirippu	0.195	0.000	46.932	88.804	22.425	73.917
156	Red	Pk-21	0.152	0.001	62.841	88.041	25.522	59.542
157	Red	Sreyas	0.108	0.001	117.500	84.860	26.604	91.208
158	Red	Jyothi	0.065	0.000	121.818	87.786	21.716	75.792
159	Red	Mahamaga	0.065	0.001	111.750	77.608	31.903	55.792
160	Red	D1	0.152	0.001	78.864	79.898	30.261	53.292
161	Red	Airwerega	0.065	0.001	98.864	60.765	31.300	66.655
162	Red	Umamata	0.065	0.001	83.864	71.570	28.800	74.710
163	Red	Bharati	0.195	0.000	114.545	80.130	20.709	41.292
164	Red	Rohidhan-1	0.325	0.000	113.184	45.340	20.765	34.145
165	Red	Ganorhibuna	0.325	0.000	60.227	43.197	28.355	40.670
166	Red	Kadamful	0.152	0.000	84.545	54.290	30.500	38.145
167	Red	Palina dhan-2	0.411	0.000	118.716	38.194	22.443	35.351

168	Red	AC-10162	0.065	0.001	83.409	76.796	24.888	64.333
169	Red	AC- 44585	0.346	0.001	85.227	48.295	29.963	48.917
170	Red	AC- 44588	0.346	0.000	54.545	83.715	35.149	186.417
171	Red	AC-44591	0.087	0.000	94.773	46.387	35.597	57.458
172	Red	AC- 44592	0.216	0.001	68.068	83.969	34.104	151.833
173	Red	AC- 44594	0.390	0.000	62.273	86.005	29.507	114.750
174	Red	AC- 44595	0.519	0.000	54.659	84.478	49.440	238.708
175	Red	AC- 44646	0.866	0.000	65.000	83.461	44.813	211.625
176	Red	AC 43658	0.130	0.000	86.364	85.496	38.843	93.708
177	Red	AC43670	0.238	0.000	80.455	88.550	59.142	287.042
178	Red	AC43676	0.281	0.000	45.341	73.664	35.522	149.125
179	Red	AC43737	0.411	0.000	52.500	88.931	36.642	166.000
180	Red	AC43738	0.606	0.000	75.341	86.768	40.933	174.958
181	Red	Alkachuri	0.135	0.000	191.989	80.540	36.005	32.360
182	Red	Amonabao	0.172	0.000	137.642	85.640	29.735	28.885
183	Red	Annapurna	0.127	0.000	157.614	83.180	51.380	46.850
184	Red	Assambiroin	0.238	0.000	106.250	88.315	42.535	33.185
185	Red	Balam	0.343	0.000	120.455	78.720	31.315	30.190
186	Red	Bambi mugai	0.230	0.000	150.114	87.550	43.270	43.715
187	Red	Barbali	0.124	0.001	93.239	84.320	41.475	29.200
188	Red	Barhasal	0.313	0.000	108.712	86.230	40.780	31.280
189	Red	Baskati	0.263	0.001	119.110	75.705	33.535	30.350
190	Red	Baula	0.317	0.001	102.917	71.385	36.800	32.470
191	Red	Bhasakalma	0.222	0.000	178.258	78.685	46.295	31.230
192	Red	Chhota dahiya	0.309	0.000	159.536	82.400	44.200	32.785
193	Red	Chingair	0.273	0.001	191.295	83.490	42.655	33.495
194	Red	Dhanigoda	0.310	0.000	126.648	79.835	33.665	31.205
195	Red	Gandhi biroin	0.268	0.000	194.970	86.325	54.695	38.690
196	Red	Gengene	0.203	0.001	132.443	82.490	47.380	35.695
197	Red	Hermanona	0.228	0.001	192.424	80.115	42.710	33.485
198	Red	Hugla	0.350	0.001	121.545	80.715	37.300	29.700
199	Red	Jool	0.225	0.000	82.415	81.670	39.010	30.855
200	Red	Kakharua	0.369	0.000	61.855	82.645	43.195	31.250
201	Red	Kaksal	0.258	0.000	156.799	87.790	41.295	33.175
202	Red	Kalakatki	0.461	0.001	195.364	86.285	40.210	33.510
203	Red	Kalokumara	0.323	0.001	170.909	81.790	38.795	39.300
204	Red	Karaga goda	0.261	0.000	189.640	80.475	42.800	40.180
205	Red	Karhani	0.548	0.000	128.409	79.910	41.430	41.200
206	Red	Karnidhan	0.220	0.001	143.939	75.885	40.290	35.185

207	Red	Katraibhog	0.368	0.000	155.341	83.005	40.085	28.955
208	Red	Kelash 1981	0.314	0.001	180.511	83.515	40.700	31.320
209	Red	Koya 4	0.177	0.000	159.280	84.690	46.395	42.695
210	Red	Koya ho baba	0.406	0.000	173.333	85.285	55.800	35.690
211	Red	Lalbora	0.372	0.000	138.182	86.240	46.595	29.310
212	Red	Langal muthi	0.408	0.000	158.182	87.415	50.010	30.200
213	Red	Likekakua	0.353	0.000	164.130	88.450	52.550	22.335
214	Red	Marchal	0.424	0.001	141.330	87.440	55.250	25.470
215	Red	Meghi	0.227	0.001	161.515	82.210	43.495	28.119
216	Red	Mornodoiga	0.376	0.001	155.455	83.495	46.505	30.365
217	Red	Motarmala	0.430	0.000	148.750	84.640	51.510	31.805
218	Red	Mugai	0.540	0.000	172.739	85.510	52.510	46.595
219	Red	Mugai	0.424	0.000	110.947	84.280	50.240	48.175
220	Red	Nagheri	0.653	0.000	119.886	86.505	48.330	39.850
221	Red	Nagheri	0.430	0.000	109.091	85.265	49.750	40.200
222	Red	Nalbora	0.367	0.000	111.591	83.505	50.210	47.725
223	Red	Neta	0.220	0.000	111.375	86.805	45.315	41.685
224	Red	Netakalani	0.274	0.001	159.795	85.450	46.205	43.420
225	Red	Nirjhara	0.322	0.000	179.758	76.400	55.565	39.855
226	Red	Palbari	0.353	0.000	147.348	82.490	35.685	40.455
227	Red	Panati	0.424	0.000	122.258	79.975	37.445	38.495
228	Red	PB140	0.419	0.000	143.864	78.550	35.510	40.875
229	Red	Rajesh	0.390	0.000	117.174	80.185	33.300	41.395
230	Red	RPHP112	0.227	0.000	168.598	81.780	31.140	35.420
231	Red	Saathi	0.349	0.000	172.764	88.715	32.965	29.875
232	Red	Setka 36	0.522	0.000	152.682	85.795	33.240	30.215
233	Red	Sugandha	0.367	0.000	170.227	82.785	39.485	32.365
234	Red	Urebanga	0.258	0.001	134.811	80.585	48.040	33.530
235	Red	Vutmari	0.221	0.000	157.604	80.920	48.195	39.795
236	Red	Geda	0.389	0.001	189.976	90.751	40.045	40.640
237	Red	Haldigundi	0.431	0.000	175.315	90.315	42.700	38.675
238	Red	Saraga debangi	0.315	0.000	189.801	89.697	35.995	39.005
239	Red	Kadara	0.289	0.001	163.629	90.800	40.660	62.830
240	Red	Sundarmadei	0.358	0.001	177.658	89.206	41.895	55.310
241	Red	Sundargada	0.291	0.000	161.472	88.017	44.170	63.495
242	Red	Kala dhusura	0.268	0.000	121.446	89.622	44.210	61.335
243	Red	Godhikhejara	0.338	0.000	199.367	90.821	43.300	59.010
244	Red	Laxmi kajal	0.415	0.000	187.031	90.932	40.710	50.190
245	Red	Mugei	0.223	0.001	131.560	88.900	43.300	52.915

246	Red	Mugi	0.554	0.000	100.080	89.528	44.785	55.320
247	Red	Jhumpuri malata	0.771	0.000	108.032	88.900	42.205	58.480
248	Red	Sal jhantri	0.518	0.000	143.321	90.163	43.215	61.325
249	Red	Nathmohan	0.450	0.001	142.499	89.685	44.785	62.435
250	Red	Hundamakara	0.242	0.001	172.295	90.324	43.315	63.440
251	Red	Dhoba kakiri	0.355	0.001	176.513	90.577	41.680	66.490
252	Red	Boula	0.328	0.001	171.101	89.930	41.195	69.688
253	Red	Sundarbhojna	0.290	0.000	160.938	90.450	41.160	70.210
254	Red	Kuliha	0.384	0.001	195.403	89.800	41.308	71.300
255	Red	Hajirimala	0.319	0.000	115.595	90.806	42.890	58.715
256	Red	Nadiarasa	0.259	0.000	150.948	89.755	43.270	59.710
257	Red	Kalanpati	0.482	0.001	133.851	89.680	44.495	60.410
258	Red	Majhi	0.258	0.001	131.805	89.807	40.210	61.235
259	Red	Kalajeri	0.397	0.001	172.255	89.912	41.540	62.320
260	Red	Majhi	0.407	0.000	163.048	89.168	40.190	61.360
261	Red	Sunakathi	0.289	0.001	147.269	89.767	41.468	54.510
262	Red	Luna	0.139	0.000	182.137	85.362	49.885	45.410
263	Red	Abhirman	0.151	0.001	176.592	87.212	52.705	49.330
264	Red	Kalakanhu	0.229	0.001	127.481	88.441	53.365	42.610
265	Red	Pologada	0.359	0.001	120.098	87.364	48.860	41.195
266	Red	Majhi	0.553	0.000	113.643	86.724	50.760	40.605
267	Red	Rajapateni	0.449	0.000	157.014	80.727	51.400	46.805
268	Red	Dalipahata	0.370	0.000	125.690	86.575	52.350	48.875
269	Red	Kadara	0.431	0.001	167.602	80.602	49.245	50.925
270	Red	Nadiarasa	0.473	0.001	181.206	82.651	49.990	52.765
Mean			0.2493	0.00033	82.67	53.50	26.33	45.35
CV %			11.23	12.42	6.38	5.52	5.63	6.72
$LSD_{5\%}$			0.0486	0.00009	6.713	3.712	1.622	6.81

Supplementary Table S2. Estimation of genetic diversity parameters based on 131 SSR marker loci in a panel containing 117 rice germplasm lines

Sl. No	Marker	No. of Alleles	Range of amplicon (bp)	Major allele frequency	Gene diversity	Heterozygosity	PIC	Inbreeding coefficient (f)
1	RM6275	4	140-160	0.714	0.455	0.060	0.419	0.870
2	RM50	4	190-205	0.410	0.689	0.026	0.632	0.963
3	RM222	4	210-250	0.620	0.567	0.026	0.527	0.955
4	RM247	5	140-200	0.496	0.594	0.068	0.514	0.886
5	RM328	3	185-200	0.564	0.582	0.000	0.515	1.000
6	RM337	6	155-400	0.440	0.673	0.120	0.617	0.823
7	RM340	5	100-220	0.714	0.452	0.094	0.413	0.794
8	RM472	3	290-410	0.517	0.508	0.094	0.387	0.816
9	RM506	3	120-130	0.692	0.452	0.137	0.386	0.700
10	RM1812	3	130-140	0.444	0.608	0.000	0.525	1.000
11	RM3701	4	160-260	0.679	0.481	0.479	0.427	0.009
12	RM6947	3	150-160	0.880	0.217	0.000	0.203	1.000
13	RM14978	3	240-250	0.410	0.638	0.000	0.561	1.000
14	RM18776	3	175-200	0.842	0.273	0.026	0.247	0.907
15	RM22034	3	75-85	0.915	0.159	0.000	0.150	1.000
16	RM24161	4	270-290	0.530	0.620	0.120	0.559	0.808
17	RM223	5	110-170	0.654	0.536	0.060	0.504	0.889
18	RM440	5	160-210	0.415	0.691	0.256	0.637	0.632
19	RM201	4	150-160	0.470	0.578	0.034	0.486	0.941
20	RM216	4	145-160	0.521	0.636	0.120	0.581	0.813
21	RM258	3	140-150		0.653	0.000	0.578	1.000

				0.385				
22	RM286	4	100-130	0.462	0.637	0.094	0.566	0.853
23	RM3735	4	135-500	0.329	0.727	0.957	0.676	-0.313
24	RM1347	3	100-110	0.513	0.568	0.000	0.477	1.000
25	RM7571	3	130-140	0.705	0.441	0.009	0.379	0.981
26	RM14723	4	220-250	0.496	0.642	0.205	0.581	0.683
27	RM103	3	255-330	0.491	0.560	0.761	0.462	-0.355
28	RM315	3	135-140	0.863	0.240	0.000	0.218	1.000
29	RM225	3	135-150	0.517	0.550	0.179	0.451	0.676
30	RM486	3	130-140	0.645	0.475	0.111	0.384	0.768
31	RM256	3	110-150	0.722	0.410	0.060	0.339	0.855
32	RM1113	3	150-180	0.662	0.463	0.060	0.377	0.872
33	RM3423	3	125-140	0.496	0.572	0.000	0.479	1.000
34	RM6100	3	170-180	0.444	0.640	0.034	0.565	0.947
35	RM590	3	140-150	0.718	0.439	0.068	0.391	0.845
36	RM5793	3	115-130	0.628	0.530	0.009	0.468	0.984
37	RM405	3	100-110	0.675	0.491	0.000	0.442	1.000
38	RM547	5	190-300	0.470	0.575	0.162	0.484	0.720
39	RM7364	5	180-250	0.620	0.573	0.171	0.540	0.704
40	RM205	3	130-180	0.611	0.540	0.026	0.473	0.953
41	RM167	4	130-180	0.697	0.471	0.103	0.429	0.784
42	RM229	4	120-140	0.355	0.708	0.128	0.653	0.820
43	RM20A	3	230-240	0.628	0.530	0.009	0.468	0.984

44	RM235	5	100-145	0.397	0.719	0.179	0.671	0.752
45	RM7003	4	100-110	0.667	0.501	0.085	0.452	0.831
46	RM5436	4	155-190	0.432	0.621	0.051	0.545	
47	RM25181	5	130-160	0.389	0.710	0.162	0.661	
48	RM469	3	100-110	0.628	0.519	0.043	0.450	
49	RM6547	3	155-165	0.863	0.245	0.017	0.231	
50	RM152	4	145-155	0.496	0.635	0.017	0.572	
51	RM148	2	140-150	0.675	0.439	0.085	0.342	
52	RM421	3	250-260	0.462	0.631	0.000	0.556	
53	RM2634	3	100-120	0.380	0.658	0.026	0.583	
54	RM248	4	75-115	0.338	0.735	0.120	0.687	
55	RM7179	5	50-250	0.321	0.767	0.359	0.728	
56	RM215	3	155-165	0.615	0.492	0.017	0.394	
57	RM324	4	220-260	0.543	0.634	0.145	0.589	
58	RM317	3	150-160	0.718	0.410	0.000	0.332	
59	RM174	3	230-270	0.504	0.623	0.068	0.553	
60	RM556	3	190-210	0.838	0.285	0.034	0.266	
61	RM257	4	130-155	0.402	0.666	0.239	0.598	
62	RM502	3	260-265	0.803	0.325	0.000	0.286	
63	RM331	4	95-115	0.496	0.659	0.051	0.607	
64	RM403	4	110-130	0.594	0.571	0.085	0.515	
65	RM309	3	180-190	0.714	0.443	0.026	0.394	
66	RM6641	3	140-145	0.556	0.590	0.000	0.523	

67	RM3	3	110-120	0.393	0.661	0.034	0.587	
68	RM594	3	300-320	0.577	0.565	0.009	0.494	
69	RM3392	4	160-180	0.517	0.612	0.111	0.544	
70	RM1278	3	135-150	0.795	0.343	0.068	0.312	
71	RM168	3	95-125	0.620	0.514	0.145	0.435	
72	RM3375	3	190-200	0.573	0.569	0.034	0.498	
73	RM282	3	140-150	0.726	0.434	0.000	0.393	
74	RM26632	4	450-550	0.359	0.701	0.154	0.644	
75	RM1341	3	170-190	0.603	0.536	0.026	0.461	
76	RM4112	3	160-170	0.483	0.625	0.162	0.552	
77	RM20377	4	300-380	0.765	0.377	0.068	0.332	
78	RM210	5	130-180	0.355	0.736	0.701	0.689	
79	RM218	4	130-160	0.590	0.581	0.034	0.529	
80	RM494	5	130-180	0.368	0.722	0.026	0.675	
81	RM336	5	105-160	0.393	0.712	0.094	0.663	
82	RM3475	4	135-160	0.444	0.657	0.043	0.591	
83	RM480	4	190-210	0.526	0.625	0.026	0.568	
84	RM566	4	150-200	0.432	0.651	0.009	0.584	
85	RM11701	3	210-230	0.641	0.472	0.000	0.376	
86	RM220	6	85-130	0.359	0.746	0.188	0.704	
87	RM488	6	155-200	0.321	0.750	0.188	0.709	
88	RM6374	6	130-160	0.329	0.775	0.077	0.741	
89	RM233	5	130-160	0.355	0.728	0.231	0.681	

90	RM112	3	130-135	0.872	0.227	0.000	0.208	
91	RM13600	4	105-130	0.483	0.661	0.094	0.606	
92	RM495	3	145-165	0.607	0.554	0.034	0.494	
93	RM493	7	180-250	0.282	0.814	0.556	0.789	
94	RM444	5	180-240	0.329	0.770	0.145	0.734	
95	RM468	3	210-220	0.765	0.386	0.026	0.353	
96	RM6054	3	120-130	0.923	0.145	0.017	0.140	
97	RM509	3	165-170	0.752	0.403	0.000	0.366	
98	RM5638	6	190-240	0.603	0.597	0.137	0.567	
99	RM8044	6	240-300	0.282	0.762	0.231	0.723	
100	RM8271	5	180-250	0.410	0.722	0.128	0.679	
101	RM171	4	380-420	0.521	0.632	0.060	0.575	
102	RM16686	3	90-100	0.410	0.656	0.000	0.582	
103	RM434	4	250-280	0.564	0.597	0.026	0.539	
104	RM6091	4	70-80	0.812	0.325	0.000	0.305	
105	RM209	4	145-175	0.538	0.614	0.000	0.554	
106	RM245	4	145-155	0.590	0.573	0.000	0.515	
107	RM1089	4	210-260	0.427	0.633	0.068	0.560	
108	RM228	4	110-170	.632	0.537	0.197	0.485	
109	RM401	3	250-300	0.765	0.384	0.060	0.347	
110	RM11	3	140-160	0.466	0.580	0.009	0.489	
111	RM3351	3	170-190	0.590	0.510	0.000	0.410	
112	RM5749	3	130-160	0.585	0.499	0.026	0.390	

113	RM335	2	100-110	0.731	0.393	0.077	0.316	
114	RM144	3	200-210	0.603	0.498	0.162	0.397	
115	RM300	3	125-145	0.863	0.243	0.017	0.226	
116	RM1132	4	90-125	0.359	0.725	0.034	0.675	
117	RM400	4	210-260	0.376	0.714	0.479	0.662	
118	RM471	3	100-120	0.795	0.345	0.000	0.315	
119	RM243	3	120-140	0.581	0.552	0.017	0.475	
120	RM467	3	200-210	0.573	0.569	0.000	0.498	
121	RM564	4	250-300	0.453	0.601	0.103	0.518	
122	RM8007	3	130-150	0.769	0.382	0.000	0.350	
123	RM441	4	160-200	0.474	0.630	0.556	0.560	
124	RM518	3	150-170	0.538	0.539	0.000	0.439	
125	RM253	4	130-170	0.551	0.597	0.085	0.533	
126	RM274	3	75-80	0.658	0.484	0.000	0.411	
127	RM242	4	200-240	0.581	0.585	0.017	0.531	
128	RM3231	4	170-550	0.346	0.704	0.650	0.646	
129	RM5687	4	160-500	0.419	0.687	0.650	0.630	
130	RM5626	3	165-180	0.585	0.511	0.726	0.411	
131	RM452	3	240-250	0.462	0.623	0.000	0.545	
	Mean	3.7099	140-160	0.560	0.556	0.111	0.496	

Supplementary Table S3. The inferred ancestry value and population structure of individual member with their antioxidants classification in the panel population at K=2.

Sl. No.	Accession No./ Vernaular name of germplasm line	Inferred ancestry value at K=2		Structure Group	Antioxidants content in each germplasm line
		Q1	Q2		
1	AC5993	0.994	0.006	SP1	Very low
2	AC6170	0.994	0.006	SP1	Low
3	AC6023	0.962	0.038	SP1	High Peroxidase
4	AC6172	0.933	0.067	SP1	Low
5	AC6027	0.446	0.554	Admix	Very low
6	AC9006	0.992	0.008	SP	Very low
7	AC9021	0.982	0.018	SP1	Very low
8	AC9028	0.93	0.07	SP1	High Peroxidase
9	AC9030	0.994	0.006	SP1	High Catalase
10	AC9035	0.99	0.01	SP1	High Peroxidase
11	AC9038	0.998	0.002	SP1	Very low
12	AC9043	0.997	0.003	SP1	Low
13	AC9044A	0.993	0.007	SP1	Very low
14	AC20920	0.994	0.006	SP1	High DPPH
15	AC20907	0.996	0.004	SP1	High DPPH
16	AC20845	0.996	0.004	SP1	High Peroxidase
17	AC20770	0.997	0.003	SP1	High DPPH
18	AC20627	0.997	0.003	SP1	High DPPH
19	AC20686	0.997	0.003	SP1	Very low

20	AC20664	0.996	0.004	SP1	Low
21	AC20614	0.998	0.002	SP1	High DPPH
22	Jhagrikartik	0.982	0.018	SP1	Very low
23	Dadghani	0.983	0.017	SP1	Very low
24	Shayam	0.451	0.549	Admix	High Catalase
25	Basumati-B	0.507	0.493	Admix	Low
26	Bharati	0.998	0.002	SP1	High DPPH,
27	Joha	0.997	0.003	SP1	Very low
28	Adira-1	0.726	0.274	Admix	Medium
29	Adira-2	0.997	0.003	SP1	High DPPH
30	Adira-3	0.791	0.209	Admix	High DPPH
31	PK6	0.988	0.012	SP1	High DPPH
32	Vachaw	0.988	0.012	SP1	High DPPH
33	Kozhivalan	0.997	0.003	SP1	High DPPH
34	Marathondi	0.641	0.359	Admix	High DPPH
35	Ezhoml-2	0.998	0.002	SP1	High DPPH
36	Jyothi	0.998	0.002	SP1	High DPPH
37	Kantakapura	0.998	0.002	SP1	High DPPH
38	Kantakaamala	0.739	0.261	Admix	High DPPH
39	Kapanthi	0.451	0.549	Admix	High DPPH
40	Karpurkanti	0.482	0.518	Admix	Very low
41	Kathidhan	0.879	0.121	SP1	High DPPH
42	Kundadhan	0.994	0.006	SP1	High TPC &DPPH
43	Champaeisiali	0.995	0.005	SP1	High DPPH

44	Latamahu	0.996	0.004	SP1	Low
45	Latachaunri	0.993	0.007	SP1	High DPPH &Peroxidase
46	AC10608	0.994	0.006	SP1	High Peroxidase
47	AC10187	0.934	0.066	SP1	High DPPH
48	AC10162	0.943	0.057	SP1	High DPPH
49	AC7282	0.413	0.587	Admix	High Peroxidase
50	AC7269	0.995	0.005	SP1	Very low
51	AC7134	0.809	0.191	SP1	Low
52	AC7008	0.997	0.003	SP1	Low
53	AC9093	0.997	0.003	SP1	High Catalase
54	AC9090	0.992	0.008	SP1	Very low
55	AC9076A	0.99	0.01	SP1	Low
56	AC9065	0.992	0.008	SP1	Low
57	AC9063	0.99	0.01	SP1	High TPC
58	AC9058	0.998	0.002	SP1	Low
59	AC9053A	0.826	0.174	SP1	High Peroxidase
60	AC9050	0.995	0.005	SP1	Low
61	AC9005	0.988	0.012	SP1	Low
62	AC20389	0.921	0.079	SP1	Low
63	AC20371	0.988	0.012	SP1	Low
64	AC20423	0.994	0.006	SP1	Low
65	AC20362	0.965	0.035	SP1	Low
66	AC20328	0.985	0.015	SP1	Low
67	AC20317	0.995	0.005	SP1	Low

68	AC20282	0.87	0.13	SP1	High CUPRAC, Cata, TPC, DPPH & FRAC
69	AC20246	0.886	0.114	SP1	High CUPRAC,FRAC &DPPH
70	AC20347	0.905	0.095	SP1	Low
71	Palinadhan-1	0.546	0.454	Admix	High DPPH
72	Chatuimuchi	0.384	0.616	Admix	Very low
73	Uttarbangalocal-9	0.871	0.129	SP1	Very low
74	Gochi	0.911	0.089	SP1	High DPPH
75	Sugandha-2	0.418	0.582	Admix	Very low
76	Jhingesal	0.997	0.003	SP1	High Catatalase
77	Cheruvirippu	0.993	0.007	SP1	High DPPH
78	Mahamaga	0.975	0.025	SP1	High DPPH
79	Jaya	0.99	0.01	SP1	Low
80	D1	0.922	0.078	SP1	High DPPH
81	Pk-21	0.983	0.017	SP1	High DPPH
82	Gandhakasala	0.411	0.589	Admix	Very low
83	Sreyas	0.995	0.005	SP1	High DPPH
84	Gondiachampeisiali	0.995	0.005	SP1	High DPPH
85	Chinamal	0.996	0.004	SP1	Low
86	Magra	0.996	0.004	SP1	Very low
87	Landi	0.997	0.003	SP1	High DPPH
88	Lalgundi	0.991	0.009	SP1	Very low
89	Balisaralaktimachi	0.995	0.005	SP1	Very low
90	Laxmibilash	0.683	0.317	Admix	Low
91	Kaniar	0.988	0.012	SP1	High DPPH

92	Kanakchampa	0.989	0.011	SP1	Very low
93	Magura-s	0.995	0.005	SP1	Very low
94	AC44603	0.049	0.951	SP2	Low
95	AC44585	0.008	0.992	SP2	High Peroxidase
96	AC44598	0.01	0.99	SP2	Low
97	AC44592	0.994	0.006	SP1	High DPPH
98	AC44646	0.994	0.006	SP1	High Cata, DPPH,FRAC &CUPRAC
99	AC44604	0.962	0.038	SP1	Medium
100	AC44597	0.003	0.997	SP2	Medium
101	AC44638	0.003	0.997	SP2	Low
102	AC44595	0.013	0.987	SP2	High CUPRAC,FRAC &DPPH,
103	AC44588	0.006	0.994	SP2	High CUPRAC,DPPH &FRAC
104	AC44591	0.037	0.963	SP2	Low
105	AC44594	0.01	0.99	SP2	High DPPH
106	AC43737	0.004	0.996	SP2	High DPPH & CUPRAC
107	AC43660	0.006	0.994	SP2	High Cata, DPPH,FRAC,CUPRAC
108	AC43732	0.022	0.978	SP2	High Cata,DPPH & CUPRAC
109	AC43661	0.004	0.996	SP2	Low
110	AC43738	0.008	0.992	SP2	High Cata & CUPRAC
111	AC43669	0.004	0.996	SP2	High DPPH,
112	AC43663	0.01	0.99	SP2	High DPPH
113	AC43658	0.004	0.996	SP2	High DPPH
114	AC43662	0.007	0.993	SP2	Low
115	AC43670	0.003	0.997	SP2	High DPPH &CUPRAC

116	AC43675	0.002	0.998	SP2	High DPPH
117	AC43676	0.003	0.997	SP2	Medium

Supplementary Table S4. Marker-trait associations with antioxidant traits in the panel population detected by the model GLM at p<0.01.

Sl.No	Trait	Marker	marker_F	marker_p	marker_Rsq
1	Catalase	RM1341	9.99747	0.00204	0.08016
2	Catalase	RM112	8.93649	0.00346	0.07169
3	Catalase	RM6054	7.68545	0.00656	0.06285
4	Catalase	RM3231	10.55577	0.00154	0.08424
5	Peroxidase	RM6275	8.88281	0.00355	0.07158
6	TPC	RM247	9.25783	0.00294	0.07648
7	TPC	RM337	7.4849	0.00728	0.06279
8	TPC	RM3701	10.51241	0.00158	0.08593
9	TPC	RM14723	8.42893	0.00448	0.07013
10	TPC	RM1113	8.96849	0.00341	0.07428
11	TPC	RM3423	7.36329	0.00775	0.06183
12	TPC	RM405	9.68297	0.00237	0.07903
13	TPC	RM7179	10.78025	0.00138	0.08792
14	TPC	RM13600	11.44114	9.99E-04	0.09202
15	TPC	RM243	7.08581	0.00896	0.05964
16	DPPH	RM247	10.72025	0.00142	0.08855
17	DPPH	RM3701	11.90813	7.99E-04	0.09738
18	DPPH	RM5793	11.8482	8.22E-04	0.09694
19	DPPH	RM167	14.36149	2.49E-04	0.11509
20	DPPH	RM6547	10.03941	0.00199	0.0827

21	DPPH	RM2634	8.67263	0.00395	0.07227
22	DPPH	RM248	6.90645	0.00984	0.05894
23	DPPH	RM7179	16.32289	1.00E-04	0.12875
24	DPPH	RM309	8.58406	0.00414	0.0722
25	DPPH	RM6374	9.20723	0.00302	0.07703
26	DPPH	RM13600	9.40651	0.00273	0.0779
27	DPPH	RM468	11.55847	9.43E-04	0.09401
28	FRAP	RM247	9.11231	0.00317	0.06781
29	FRAP	RM3701	12.35495	6.44E-04	0.08946
30	FRAP	RM223	7.31149	0.00795	0.05478
31	FRAP	RM5793	9.3855	0.00276	0.06968
32	FRAP	RM167	9.81615	0.00223	0.07261
33	FRAP	RM6547	8.50627	0.0043	0.06309
34	FRAP	RM309	8.01123	0.00554	0.06018
35	FRAP	RM13600	7.99722	0.00558	0.05957
36	FRAP	RM468	8.51023	0.00429	0.06312
37	CUPRAC	RM247	10.17572	0.00186	0.07494
38	CUPRAC	RM3701	14.56812	2.26E-04	0.10344
39	CUPRAC	RM315	7.84852	0.00603	0.05896
40	CUPRAC	RM235	10.18416	0.00185	0.07436
41	CUPRAC	RM148	7.69767	0.00652	0.0579
42	CUPRAC	RM421	7.19824	0.00845	0.05438

Supplementary Table S5. Marker-trait associations with antioxidant traits, cataase, peroxidase, TPC, DPPH, FRAP and CUPRAC in the panel population detected by the model MLM at $p<0.01$.

Sl.No.	Trait	Marker	F	p	MarkerR2
1	Catalase	RM1341	7.8566	0.006	0.07179
2	Catalase	RM3231	8.16013	0.00514	0.07457
3	DPPH	RM247	10.07384	0.00196	0.09162
4	DPPH	RM3701	11.09733	0.00118	0.10093
5	DPPH	RM13600	6.88723	0.00994	0.06264
6	FRAP	RM247	7.14597	0.00868	0.06551
7	FRAP	RM3701	8.98419	0.00338	0.08236
8	FRAP	RM309	7.35763	0.00777	0.06745
9	CUPRAC	RM3701	9.65365	0.00241	0.08678
10	CUPRAC	RM18776	14.00182	2.94E-04	0.12586
11	CUPRAC	RM235	10.03931	0.00199	0.09024
12	CUPRAC	RM148	7.03523	0.0092	0.06324
13	CUPRAC	RM220	7.37227	0.00771	0.06627
